# Finite Difference Method for Time-Space Fractional Advection–Diffusion Equations with Riesz Derivative

**DOI:** 10.3390/e20050321

**Published:** 2018-04-26

**Authors:** Sadia Arshad, Dumitru Baleanu, Jianfei Huang, Maysaa Mohamed Al Qurashi, Yifa Tang, Yue Zhao

**Affiliations:** 1 The State Key Laboratory of Scientific and Engineering Computing (LSEC), The Institute of Computational Mathematics and Scientific/Engineering Computing (ICMSEC), Academy of Mathematics and Systems Science, Chinese Academy of Sciences, Beijing 100190, China; 2 COMSATS Institute of Information Technology, Lahore 54500, Pakistan; 3 Department of Mathematics, Cankaya University, Ankara 06530, Turkey; 4 Institute of Space Sciences, Magurele-Bucharest 077125, Romania; 5 College of Mathematical Sciences, Yangzhou University, Yangzhou 225002, China; 6 Department of Mathematics, King Saud University, P.O. Box 22452, Riyadh 11495, Saudi Arabia; 7 School of Mathematical Sciences, University of Chinese Academy of Sciences, Beijing 100049, China

**Keywords:** fractional advection dispersion equation, riesz derivative, caputo derivative, trapezoidal formula

## Abstract

In this article, a numerical scheme is formulated and analysed to solve the time-space fractional advection–diffusion equation, where the Riesz derivative and the Caputo derivative are considered in spatial and temporal directions, respectively. The Riesz space derivative is approximated by the second-order fractional weighted and shifted Grünwald–Letnikov formula. Based on the equivalence between the fractional differential equation and the integral equation, we have transformed the fractional differential equation into an equivalent integral equation. Then, the integral is approximated by the trapezoidal formula. Further, the stability and convergence analysis are discussed rigorously. The resulting scheme is formally proved with the second order accuracy both in space and time. Numerical experiments are also presented to verify the theoretical analysis.

## 1. **Introduction**

The concepts of fractional calculus and entropy are becoming more popular for analyzing the dynamics of complex systems. The idea of entropy was introduced in the field of thermodynamics by Clausius (1862) and Boltzmann (1896) and was later applied by Shannon (1948) and Jaynes (1957) in information theory. Recently, more general entropy measures have being proposed for application in several types of complex systems due to the relaxation of the additivity axiom. The concept of entropy for analyzing the dynamics of multi-particle systems with integer and fractional order behavior was proposed in [1]. The entropy production rate for the fractional diffusion process was calculated in [2]. In [3] it has been shown that the total spectral entropy can be used as a measure of the information content in a fractional order model of anomalous diffusion.

Fractional calculus has been applied to almost every field of science, engineering, and mathematics during the last decades [4,5,6,7,8]. Particularly fractional calculus has significant impact in the fields of viscoelasticity and rheology, physics, electrical engineering, electrochemistry, signal and image processing, biology, biophysics and bioengineering, mechanics, mechatronics, and control theory. Fractional calculus is indeed a worthwhile mathematical tool that can undertake more than integer calculus. The monographs authored by Samko, Kilbas, Marichev [9], Podlubny [10] and Kilbas, Srivastava, Trujillo [7] have been helpful in understanding the theory and applications of fractional differential equations.

Numerous numerical methods have been proposed for solving the time-fractional differential equations. In this paper, we convert the fractional differential equation into the equivalent integral equation. Then, fractional trapezoidal formula is used to approximate fractional integral which has second-order accuracy [11,12]. Early in 1993, Tang [13] presented a finite difference method for the numerical solution of the partial integro-differential equations with a weakly singular kernel based on the product trapezoidal formula. Chen et al. [14] proposed fractional trapezoidal rule (FTR) type difference scheme by combining the second order difference quotient for spatial discretization and the FTR alternating direction implicit method in the time stepping for a two-dimensional fractional evolution equation. Chen et al. [15] derived a fractional trapezoidal rule type difference scheme for fractional order integro-differential equation with second order accuracy both in temporal and spatial directions. Recently, a finite difference scheme has been proposed in [16] to solve time-space fractional diffusion equation of second-order accuracy in both time and space by employing trapezoidal rule. Numerical schemes for linear and nonlinear time-space fractional diffusion equations were constructed in [17] using the trapezoidal formula for temporal approximation and the centred difference approximation for the spatial Riesz fractional derivative. Several numerical schemes have been proposed to approximate Riesz fractional derivative based on numerical methods to approximate Riemann–Liouville derivative such as standard Grünwald–Letnikov formula (first-order accuracy), shifted Grünwald–Letnikov formula (first-order accuracy) [18], L-2 approximation method [19] (first-order accuracy), spline interpolation method [20] (second-order accuracy), weighted and shifted Grünwald–Letnikov formulas [21] (second and third-order accuracy), fractional average central difference formula [22] (second and fourth-order accuracy). It is worth mentioning that high-order algorithms for Riemann–Liouville derivatives were first proposed by Lubich [23], however, the high order algorithms for Riesz derivatives were constructed by Ding and Li [22,24,25].

Many researchers studied the fractional advection–dispersion equation (ADE) recently. Fractional ADE is used for the description of transport dynamics in the complex systems which are controlled by the anomalous diffusion and the non-exponential relaxation patterns [26]. The fractional ADE is also used in groundwater hydrology research to model the transport of passive tracers carried by the fluid flow in a porous medium [27]. Our aim is to investigate the time-space fractional ADE. Time nonlocality deals with memory effects, whereas space nonlocality describes the long-range interaction. The fundamental idea is that fractional order models convey more information about the underlying structure and dynamics of complex systems. Total Shannon spectral entropy for the case of anomalous diffusion governed by a fractional order diffusion equation generalized in space and in time is calculated in [3] as it can be used as a measure of the information content in a fractional order model of anomalous diffusion. This fractional order representation of the continuous time, random walk model of diffusion gives a spectral entropy minimum for normal (i.e., Gaussian) diffusion with surrounding values leading to greater values of spectral entropy. Povstenko et al. [28] examined the fundamental solutions to space-time fractional diffusion equation with mass absorption (mass release) in the case of axial symmetry. Liu et al. [29] considered time fractional ADE and the solution was obtained using variable transformation, Mellin and Laplace transforms, and H-functions. Povstenko and Kyrylych [30] discussed two different generalizations of the space-time fractional advection–diffusion equation. They studied the fundamental solutions to the corresponding Cauchy and source problems for one spatial variable using Laplace transform and Fourier transform with respect to time and spatial coordinate, respectively. Huang and Liu [31] also considered time-space fractional ADE and obtained the solution in terms of Green functions. Meerschaert et al. [18] proposed numerical methods to solve the one-dimensional space fractional ADE with variable coefficients on a finite domain. Tripathi et al. [32] investigated the approximate analytical solution of fractional order nonlinear diffusion equations by using the homotopy analysis method. Momani et al. [33] developed a reliable algorithm using the Adomian decomposition method to construct a numerical solution for the time-space fractional ADE. Liu et al. [34] proposed an approximation of the Lëvy–Feller advection–dispersion process by employing a random walk and finite difference methods. Finite difference methods [35], finite element methods [36], finite volume methods [37], homotopy perturbation methods [38] and spectral methods [39,40] are also employed to approximate the fractional ADE. Furthermore, recent advances in numerical linear algebra had a substantial impact on designing efficient methods for the solution of the resulting linear systems which are dense but whose computational cost can be essentially reduced to O(Nlog(N)) where *N* is the size of the underlying coefficient matrix (see [41,42,43,44] and references therein). In this article, we construct a numerical scheme for the time-space fractional ADE by transforming the fractional differential equations into equivalent Volterra integral equations. As it is known that numerical methods for an integral equation have better numerical stability over the schemes designed for equivalent differential equation. Also the numerical methods for an integral equation can be constructed based on the weaker smoothness requirement than that for the differential equation. To the best of our knowledge, all of the other higher order methods are proposed based on the discretizations for fractional derivative directly.

This paper is organized as follows. In Section 2, some useful notations and auxiliary lemmas are introduced. In Section 3, the fractional trapezoidal scheme is derived combined with the second-order fractional weighted and shifted Grünwald–Letnikov formula for the approximation of the Riesz derivative. Section 4 is devoted to the study of the stability and convergence of the proposed scheme. Some numerical experiments are presented to verify the efficiency of our theoretical results in Section 5. The last section concludes our work.

## 2. Preliminaries

**Definition** **1.** *The γ(n−1<γ<n) order left and right Riemann–Liouville fractional derivatives of the function f on [a,b] are given by:*

*Left Riemann–Liouville fractional derivative:*

$${}_{a}D_{x}^{\gamma }f\left(x\right)=\frac{1}{\Gamma (n-\gamma )}\frac{d^{n}}{dx^{n}}\int_{a}^{x}{\left(x-s\right)}^{n-\gamma -1}f\left(s\right)ds,$$

*Right Riemann–Liouville fractional derivative:*

$${}_{x}D_{b}^{\gamma }f\left(x\right)=\frac{{\left(-1\right)}^{n}}{\Gamma (n-\gamma )}\frac{d^{n}}{dx^{n}}\int_{x}^{b}{\left(x-s\right)}^{n-\gamma -1}f\left(s\right)ds.$$

**Definition** **2.** *The Caputo fractional derivative of order 0<α<1 of the function f on [a,b] is defined by:*

$${}^{c}D_{t}^{\alpha }f\left(x\right)=\frac{1}{\Gamma (1-\alpha )}\int_{a}^{x}{\left(x-s\right)}^{\alpha -1}\frac{d}{dx}f\left(s\right)ds.$$

**Definition** **3.** *The Riemann–Louville fractional integral of order α>0 of the function f on [a,b] is defined by:*

$$I^{\alpha }f\left(x\right)=\frac{1}{\Gamma (\alpha )}\int_{a}^{x}{\left(x-s\right)}^{\alpha -1}f\left(s\right)ds.$$

In this paper, we will consider the following time-space fractional ADE

(1) $${}^{c}D_{t}^{\alpha }u\left(x,t\right)=K_{\beta_{1}}\frac{\partial^{\beta_{1}}}{{\partial |x|}^{\beta_{1}}}u\left(x,t\right)+K_{\beta_{2}}\frac{\partial^{\beta_{2}}}{{\partial |x|}^{\beta_{2}}}u\left(x,t\right)+f\left(x,t\right),\;\;\;\;\;0<x<L,0<t\leq T,$$

with the initial conditions

(2) u(x,0)=φ(x),0≤x≤L,

and the Dirichlet boundary conditions

(3) u(0,t)=u(L,t)=0,0≤t≤T,

where 0<α<1, $0<\beta_{1}<1,\;1<\beta_{2}\leq 2,K_{\beta_{1}}\geq 0,\;K_{\beta_{2}}>0$ and ${}^{c}D_{t}^{\alpha }$ is the Caputo fractional derivative. In addition, $\frac{\partial^{\beta_{1}}}{{\partial |x|}^{\beta_{1}}}$ and $\frac{\partial^{\beta_{2}}}{{\partial |x|}^{\beta_{2}}}$ are the Riesz fractional derivatives of order $\beta_{1}$ and $\beta_{2}$ respectively, defined on the domain [0,L] as follows [19]

(4) $$\frac{\partial^{\beta_{1}}}{{\partial |x|}^{\beta_{1}}}u\left(x,t\right)=-\rho_{\beta_{1}}{[}_{0}D_{x}^{\beta_{1}}u\left(x,t\right){+}_{x}D_{L}^{\beta_{1}}u\left(x,t\right)],$$

(5) $$\frac{\partial^{\beta_{2}}}{{\partial |x|}^{\beta_{2}}}u\left(x,t\right)=-\rho_{\beta_{2}}{[}_{0}D_{x}^{\beta_{2}}u\left(x,t\right){+}_{x}D_{L}^{\beta_{2}}u\left(x,t\right)],$$

where

$$\rho_{\beta_{1}}=\frac{1}{2\cos\frac{\pi \beta_{1}}{2}},\;\rho_{\beta_{2}}=\frac{1}{2\cos\frac{\pi \beta_{2}}{2}},$$

and

$${}_{0}D_{x}^{\beta_{1}}u\left(x,t\right)=\frac{1}{\Gamma (1-\beta_{1})}\frac{\partial }{\partial x}\int_{0}^{x}{\left(x-\tau \right)}^{-\beta_{1}}u\left(\tau ,t\right)d\tau ,$$

$${}_{x}D_{L}^{\beta_{1}}u\left(x,t\right)=\frac{-1}{\Gamma (1-\beta_{1})}\frac{\partial }{\partial x}\int_{x}^{L}{\left(\tau -x\right)}^{-\beta_{1}}u\left(\tau ,t\right)d\tau ,$$

$${}_{0}D_{x}^{\beta_{2}}u\left(x,t\right)=\frac{1}{\Gamma (2-\beta_{2})}\frac{\partial^{2}}{\partial x^{2}}\int_{0}^{x}{\left(x-\tau \right)}^{1-\beta_{2}}u\left(\tau ,t\right)d\tau ,$$

$${}_{x}D_{L}^{\beta_{2}}u\left(x,t\right)=\frac{1}{\Gamma (2-\beta_{2})}\frac{\partial^{2}}{\partial x^{2}}\int_{x}^{L}{\left(\tau -x\right)}^{1-\beta_{2}}u\left(\tau ,t\right)d\tau .$$

In the interval [a,b], let $x_{j}=jh,\;\left(j=0,1,\ldots M\right)$ be mesh points in space, where $h=\frac{b-a}{M}$ is the uniform spatial step size. Meerschaert and Tadjeran [18] showed that the standard Grünwald–Letnikov difference formula was often unstable for time dependent problems and they proposed the shifted Grünwald difference operators to approximate the left and right Riemann–Liouville fractional derivatives

$$A_{p}f\left(x\right)=\frac{1}{h^{\gamma }}\sum_{k=0}^{\infty }g_{k}^{\left(\gamma \right)}f\left(x-\left(k-p\right)h\right),$$

$$B_{q}f\left(x\right)=\frac{1}{h^{\gamma }}\sum_{k=0}^{\infty }g_{k}^{\left(\gamma \right)}f\left(x+\left(k-q\right)h\right),$$

that have the first order accuracy given by,

$$A_{p}f\left(x\right){=}_{-\infty }D_{x}^{\gamma }f\left(x\right)+O\left(h\right),$$

$$B_{q}f\left(x\right){=}_{x}D_{+\infty }^{\gamma }f\left(x\right)+O\left(h\right),$$

where p,q are integers, and $g_{k}^{\left(\gamma \right)}={\left(-1\right)}^{k}\left(\begin{array}{c} \gamma \\ k \end{array}\right)$. In fact, the coefficients $g_{k}^{\left(\gamma \right)}$ are the coefficients of the power series of the function ${\left(1-z\right)}^{\gamma }$,

$${\left(1-z\right)}^{\gamma }=\sum_{k=0}^{\infty }{\left(-1\right)}^{k}\left(\begin{array}{c} \gamma \\ k \end{array}\right)z^{k}=\sum_{k=0}^{\infty }g_{k}^{\left(\gamma \right)}z^{k},$$

for all |z|≤1, and they can be evaluated recursively by using the following relation

$$g_{0}^{\left(\gamma \right)}=1,\;\;g_{k}^{\left(\gamma \right)}=\left(1-\frac{\gamma +1}{k}\right)g_{k-1}^{\left(\gamma \right)},\;\;k=1,2,\ldots .$$

**Lemma** **1** ([**35**])**.** *Suppose that $0<\beta_{1}<1,$ then the coefficients $g_{k}^{\left(\beta_{1}\right)}$ satisfy*

$\left\{\begin{array}{cc} g_{0}^{\left(\beta_{1}\right)}=1,\;\;g_{1}^{\left(\beta_{1}\right)}=-\beta_{1}<0,\;\;g_{2}^{\beta_{1}}=\frac{\beta_{1}\left(\beta_{1}-1\right)}{2}<0, & \\ g_{1}^{\left(\beta_{1}\right)}<g_{2}^{\left(\beta_{1}\right)}<g_{3}^{\left(\beta_{1}\right)}\ldots <0, & \\ \sum_{k=0}^{\infty }g_{k}^{\left(\beta_{1}\right)}=0,\;\;\sum_{k=0}^{M}g_{k}^{\left(\beta_{1}\right)}>0,\;\;\;\;M\geq 1. & \end{array}\right.$

**Lemma** **2** ([**35**])**.** *Suppose that $1<\beta_{2}\leq 2,$ then the coefficients $g_{k}^{\left(\beta_{2}\right)}$ satisfy*

$\left\{\begin{array}{cc} g_{0}^{\left(\beta_{2}\right)}=1,\;\;g_{1}^{\left(\beta_{2}\right)}=-\beta_{2}<0,\;\;g_{k}^{\left(\beta_{2}\right)}=\frac{\beta_{2}\left(\beta_{2}-1\right)}{2}>0, & \\ 1\geq g_{2}^{\left(\beta_{2}\right)}\geq g_{3}^{\left(\beta_{2}\right)}\geq \ldots \geq 0, & \\ \sum_{k=0}^{\infty }g_{k}^{\left(\beta_{2}\right)}=0,\;\;\sum_{k=0}^{M}g_{k}^{\left(\beta_{2}\right)}<0,\;\;\;\;M\geq 1. & \end{array}\right.$

Tian et al. in [21] derived the following weighted shifted Grünwald difference operators based on the multi-step method

$${}_{L}D_{p,q}^{\gamma }f\left(x\right)=\frac{\gamma -2q}{2(p-q)}A_{p}f\left(x\right)+\frac{2p-\gamma }{2(p-q)}A_{q}f\left(x\right),$$

$${}_{R}D_{p,q}^{\gamma }f\left(x\right)=\frac{\gamma -2q}{2(p-q)}B_{p}f\left(x\right)+\frac{2p-\gamma }{2(p-q)}B_{q}f\left(x\right).$$

**Lemma** **3** ([**21**])**.** *Suppose that 1<γ<2, let $f\left(x\right)\in L^{1}\left(R\right),{\;}_{-\infty }D^{\gamma }f\left(x\right),D_{+\infty }^{\gamma }f\left(x\right)$ and their Fourier transforms belong to $L^{1}\left(R\right)$, then the weighted and shifted Grünwald difference operators satisfy*

$${}_{L}D_{p,q}^{\gamma }f\left(x\right){=}_{-\infty }D_{x}^{\gamma }f\left(x\right)+O\left(h^{2}\right),$$

$${}_{R}D_{p,q}^{\gamma }f\left(x\right){=}_{x}D_{+\infty }^{\gamma }f\left(x\right)+O\left(h^{2}\right),$$

*uniformly for x∈R, where p,q are integers and p≠q.*

Consider a function f(x) under the same assumptions as in Lemma 3 on the bounded interval [a,b], if f(a)=0 or f(b)=0, the function f(x) can be zero extended for x<a or x>b. In addition, then, the γ order left and right Riemann–Liouville fractional derivatives of f(x) at each point *x* can be approximated with the second order accuracy as follows

$${}_{a}D_{x}^{\gamma }f\left(x\right)=\frac{\lambda_{1}}{h^{\gamma }}\sum_{k=0}^{[\frac{x-a}{h}]+p}g_{k}^{\left(\gamma \right)}f\left(x-\left(k-p\right)h\right)+\frac{\lambda_{2}}{h^{\gamma }}\sum_{k=0}^{[\frac{x-a}{h}]+q}g_{k}^{\left(\gamma \right)}f\left(x-\left(k-q\right)h\right)+O\left(h^{2}\right),$$

$${}_{x}D_{b}^{\gamma }f\left(x\right)=\frac{\lambda_{1}}{h^{\gamma }}\sum_{k=0}^{[\frac{b-x}{h}]+p}g_{k}^{\left(\gamma \right)}f\left(x+\left(k-p\right)h\right)+\frac{\lambda_{2}}{h^{\gamma }}\sum_{k=0}^{[\frac{b-x}{h}]+q}g_{k}^{\left(\gamma \right)}f\left(x+\left(k-q\right)h\right)+O\left(h^{2}\right),$$

where $\lambda_{1}=\frac{\gamma -2q}{2(p-q)}$ and $\lambda_{2}=\frac{2p-\gamma }{2(p-q)}.$

**Lemma** **4.** *When (p,q)=(1,0) the discrete approximations for the Riemann–Liouville fractional derivatives on the domain [0,L] are*

$${}_{0}D_{x}^{\gamma }f\left(x_{j}\right)=\frac{1}{h^{\gamma }}\sum_{k=0}^{j+1}w_{k}^{\left(\gamma \right)}f\left(x_{j-k+1}\right)+O\left(h^{2}\right),$$

$${}_{x}D_{L}^{\gamma }f\left(x_{j}\right)=\frac{1}{h^{\gamma }}\sum_{k=0}^{M-j+1}w_{k}^{\left(\gamma \right)}f\left(x_{j+k-1}\right)+O\left(h^{2}\right),$$

*where*

$$w_{0}^{\left(\gamma \right)}=\frac{\gamma }{2}g_{0}^{\left(\gamma \right)},\;\;w_{k}^{\left(\gamma \right)}=\frac{\gamma }{2}g_{k}^{\left(\gamma \right)}+\frac{2-\gamma }{2}g_{k-1}^{\left(\gamma \right)},\;\;\;k\geq 1.$$

**Lemma** **5** ([**45**])**.** *Suppose that $0<\beta_{1}<1$, then the coefficients $w_{k}^{\left(\beta_{1}\right)}$ satisfy*

$\left\{\begin{array}{cc} w_{0}^{\left(\beta_{1}\right)}=\frac{\beta_{1}}{2}>0,\;\;w_{1}^{\left(\beta_{1}\right)}=\frac{2-\beta_{1}-\beta_{1}^{2}}{2}>0,\;\;w_{2}^{\left(\beta_{1}\right)}=\frac{\beta_{1}\left(\beta_{1}^{2}+\beta_{1}-4\right)}{4}<0, & \\ w_{2}^{\left(\beta_{1}\right)}<w_{3}^{\left(\beta_{1}\right)}<w_{4}^{\left(\beta_{1}\right)}<\ldots <0, & \\ \sum_{k=0}^{\infty }w_{k}^{\left(\beta_{1}\right)}=0,\;\;\sum_{k=0}^{M}w_{k}^{\left(\beta_{1}\right)}>0,\;\;\;\;M\geq 1. & \end{array}\right.$

**Lemma** **6** ([**21**])**.** *Suppose that $1<\beta_{2}\leq 2$, then the coefficients $w_{k}^{\beta_{2}}$ satisfy*

$\left\{\begin{array}{cc} w_{0}^{\left(\beta_{2}\right)}=\frac{\beta_{2}}{2}>0,\;\;w_{1}^{\left(\beta_{2}\right)}=\frac{2-\beta_{2}-\beta_{2}^{2}}{2}<0,\;\;w_{2}^{\left(\beta_{2}\right)}=\frac{\beta_{2}\left(\beta_{2}^{2}+\beta_{2}-4\right)}{4}, & \\ 1\geq w_{0}^{\left(\beta_{2}\right)}\geq w_{3}^{\left(\beta_{2}\right)}\geq w_{4}^{\left(\beta_{2}\right)}\geq \ldots \geq 0, & \\ \sum_{k=0}^{\infty }w_{k}^{\left(\beta_{2}\right)}=0,\;\;\sum_{k=0}^{M}w_{k}^{\left(\beta_{2}\right)}<0,\;\;\;\;M\geq 2. & \end{array}\right.$

**Lemma** **7** ([**46**])**.** *Suppose $u\left(t\right)\in C^{3}\left[0,T\right],\;$ for $\xi \in (t_{j},t_{j+1}),$ there exists a positive constant C>0, such that*

(6) $$\left|u\left(\xi \right)-\frac{\left(t_{j+1}-\xi \right)u\left(t_{j}\right)+\left(\xi -t_{j}\right)u\left(t_{j+1}\right)}{\tau }\right|\leq C\tau^{2}.$$

**Lemma** **8** ([**47**])**.** *Let*

(7) $$b_{n}^{\alpha }={\left(n+1\right)}^{\alpha }-n^{\alpha },n=0,1,2,\ldots ,$$

*then $b_{n}^{\alpha }={\left(n+1\right)}^{\alpha }-n^{\alpha },\left(n=0,1,2,\ldots \right)$ satisfy the following properties*

*1*. $b_{0}^{\alpha }=1,\;\;\;b_{n}^{\alpha }>0,n=0,1,2,\ldots ,$
*2*. $b_{n}^{\alpha }>b_{n+1}^{\alpha },\;\;\;n=0,1,2,\ldots ,$
*3*. *there exists a positive constant C>0, such that $\tau \leq Cb_{n}^{\alpha }\tau^{\alpha },n=1,2,3,\ldots .$*

**Lemma** **9** ([**46**])**.** *Suppose $u\left(t\right)\in C^{3}\left[0,T\right],$ then we have*

(8) $$I^{\alpha }u\left(t_{n+1}\right)-I^{\alpha }u\left(t_{n}\right)=\frac{\tau^{\alpha }}{\Gamma (\alpha +1)}\left[\left(a_{n}^{\alpha }-a_{n-1}^{\alpha }\right)u\left(t_{0}\right)+\sum_{l=0}^{n-1}\left(d_{l+1}-d_{l}\right)u\left(t_{n-l}\right)+c_{0}^{\alpha }u\left(t_{n+1}\right)\right]+R_{1},$$

*for n=0,1,2,…,N−1, where*

(9) $$\begin{array}{cc} a_{n}^{\alpha } & ={\left(n+1\right)}^{\alpha }-\frac{1}{\alpha +1}\left[{\left(n+1\right)}^{\alpha +1}-n^{\alpha +1}\right],\;\;\;n=0,1,2,\ldots , \end{array}$$

(10) $$\begin{array}{cc} c_{n}^{\alpha } & =\frac{1}{\alpha +1}\left[{\left(n+1\right)}^{\alpha +1}-n^{\alpha +1}\right]-n^{\alpha },\;\;\;n=0,1,2,\ldots , \end{array}$$

(11) $$\begin{array}{cc} d_{n}^{\alpha } & =a_{n-1}^{\alpha }+c_{n}^{\alpha }\\ & =\frac{1}{\alpha +1}\left[{\left(n+1\right)}^{\alpha +1}-2n^{\alpha +1}+{\left(n-1\right)}^{\alpha +1}\right],\;\;\;n=0,1,2,\ldots , \end{array}$$

*and $R_{1}$ depends on τ with*

(12) $$|R_{1}|\leq C\tau^{\alpha +2}\left(a_{n}^{\alpha }+c_{n}^{\alpha }\right)=C\tau^{\alpha +2}b_{n}^{\alpha }.$$

*Here, we assume $a_{-1}^{\alpha }=0,$ that is,*

(13) $$d_{0}^{\alpha }=c_{0}^{\alpha }.$$

**Lemma** **10** ([**46**])**.** *Suppose that $a_{n}^{\alpha },\;c_{n}^{\alpha },\;d_{n}^{\alpha }$ are defined by Lemma 9, then we can conclude that:*

*1*. *$a_{n}^{\alpha },\;\;\left(n=1,2,\ldots \right)$ are monotonically decreasing when n increases.*
*2*. *$c_{n}^{\alpha },\;\;\left(n=0,1,2,\ldots \right)$ are monotonically decreasing when n increases.*
*3*. *$d_{n}^{\alpha },\;\;\left(n=1,2,\ldots \right)$ are monotonically decreasing when n increases.*

## 3. Finite Difference Approximation

We define $t_{n}=n\tau ,\;n=0,1,\ldots ,N$ and $x_{j}=jh,\;j=0,1,\ldots M,$ where τ=T/N, and h=L/M are the time and space step sizes, respectively. Considering system (1)–(3) at the point $\left(x_{j},t_{n}\right)$, we have

(14) $$\left\{\begin{array}{cc} {}^{c}D_{t}^{\alpha }u\left(x_{j},t_{n}\right)=K_{\beta_{1}}\frac{\partial^{\beta_{1}}}{\partial |x_{j}{|}^{\beta_{1}}}u\left(x_{j},t_{n}\right)+K_{\beta_{2}}\frac{\partial^{\beta_{2}}}{\partial |x_{j}{|}^{\beta_{2}}}u\left(x_{j},t_{n}\right)+f\left(x_{j},t_{n}\right),\;\;\;\;\;\;1\leq j\leq M-1,\;1<n<N, & \\ u\left(x_{j},0\right)=\varphi \left(x_{j}\right),\;\;\;\;\;0\leq j\leq M, & \\ u\left(x_{0},t_{n}\right)=u\left(x_{M},t_{n}\right)=0,\;\;\;\;1\leq n\leq N. & \end{array}\right.$$

Assume that $u_{j}^{n}$ denotes the numerical approximation of $u(x_{j},t_{n})$. We can discretize the Riesz fractional derivatives $\frac{\partial^{\beta_{1}}}{{\partial |x|}^{\beta_{1}}}$ and $\frac{\partial^{\beta_{2}}}{{\partial |x|}^{\beta_{2}}}$ in truncated bounded domain as follows:

(15) $$\delta_{x}^{\beta_{1}}u\left(x_{j},t_{n}\right)=-\frac{1}{2\cos\frac{\pi \beta_{1}}{2}h^{\beta_{1}}}\left(\sum_{k=0}^{j+1}w_{k}^{\left(\beta_{1}\right)}u_{j-k+1}^{n}+\sum_{k=0}^{M-j+1}w_{k}^{\left(\beta_{1}\right)}u_{j+k-1}^{n}\right)+O\left(h^{2}\right),$$

(16) $$\delta_{x}^{\beta_{2}}u\left(x_{j},t_{n}\right)=-\frac{1}{2\cos\frac{\pi \beta_{2}}{2}h^{\beta_{2}}}\left(\sum_{k=0}^{j+1}w_{k}^{\left(\beta_{2}\right)}u_{j-k+1}^{n}+\sum_{k=0}^{M-j+1}w_{k}^{\left(\beta_{2}\right)}u_{j+k-1}^{n}\right)+O\left(h^{2}\right).$$

Let $\mu_{1}=\frac{K_{\beta_{1}}\rho_{\beta_{1}}}{h^{\beta_{1}}}$ and $\mu_{2}=\frac{K_{\beta_{2}}\rho_{\beta_{2}}}{h^{\beta_{2}}}$. Noting that $\mu_{1}>0$ and $\mu_{2}<0$ since $\rho_{\beta_{1}}=\frac{1}{2\cos\frac{\pi \beta_{1}}{2}}>0$ for $0<\beta_{1}\leq 1$ and $\rho_{\beta_{2}}=\frac{1}{2\cos\frac{\pi \beta_{2}}{2}}<0$ for $1<\beta_{2}\leq 2$. Using the approximation to the Riesz fractional derivative given in (15) and (16) into (14), we obtain

(17) $${}^{c}D_{t}^{\alpha }u\left(x_{j},t_{n}\right)=-\mu_{1}\left(\sum_{k=0}^{j+1}w_{k}^{\left(\beta_{1}\right)}u_{j-k+1}^{n}+\sum_{k=0}^{M-j+1}w_{k}^{\left(\beta_{1}\right)}u_{j+k-1}^{n}\right)-\mu_{2}\left(\sum_{k=0}^{j+1}w_{k}^{\left(\beta_{2}\right)}u_{j-k+1}^{n}+\sum_{k=0}^{M-j+1}w_{k}^{\left(\beta_{2}\right)}u_{j+k-1}^{n}\right)+f\left(x_{j},t_{n}\right),$$

where 1≤j≤M−1,1<n<N, with initial and boundary conditions discretized as follows:

(18) $$\left\{\begin{array}{cc} u_{j}\left(0\right)=u_{j}^{0},\;\;\;\;\;0\leq j\leq M, & \\ u_{0}^{n}=u_{M}^{n}=0,\;\;\;\;1\leq n\leq N. & \end{array}\right.$$

### Fractional Trapezoid Formula

In this subsection and in the sequel, the symbol *C* denotes a generic constant, whose value may be different from one line to another. Integrating both sides of (17) with respect to the time *t* from $t_{n}$ to $t_{n+1},$ and using Lemmas 4, 7 and 9, we obtain

$$\begin{array}{cc} & u_{j}^{n+1}-u_{j}^{n}\\ = & -\nu^{\alpha }c_{0}^{\alpha }\left[\mu_{1}\left(\sum_{k=0}^{j+1}w_{k}^{\left(\beta_{1}\right)}u_{j-k+1}^{n+1}\sum_{k=0}^{M-j+1}w_{k}^{\left(\beta_{1}\right)}u_{j+k-1}^{n+1}\right)-\mu_{2}\left(\sum_{k=0}^{j+1}w_{k}^{\left(\beta_{2}\right)}u_{j-k+1}^{n+1}+\sum_{k=0}^{M-j+1}w_{k}^{\left(\beta_{2}\right)}u_{j+k-1}^{n+1}\right)-R_{2}\right]\\ - & \nu^{\alpha }\left(a_{n}^{\alpha }-a_{n-1}^{\alpha }\right)\left[\mu_{1}\left(\sum_{k=0}^{j+1}w_{k}^{\left(\beta_{1}\right)}u_{j-k+1}^{0}+\sum_{k=0}^{M-j+1}w_{k}^{\left(\beta_{1}\right)}u_{j+k-1}^{0}\right)+\mu_{2}\left(\sum_{k=0}^{j+1}w_{k}^{\left(\beta_{2}\right)}u_{j-k+1}^{0}+\sum_{k=0}^{M-j+1}w_{k}^{\left(\beta_{2}\right)}u_{j+k-1}^{0}\right)-R_{2}\right]\\ - & \nu^{\alpha }\sum_{l=0}^{n-1}\left(d_{l+1}^{\alpha }-d_{l}^{\alpha }\right)\left[+\mu_{1}\left(\sum_{k=0}^{j+1}w_{k}^{\left(\beta_{1}\right)}u_{j-k+1}^{n-l}+\sum_{k=0}^{M-j+1}w_{k}^{\left(\beta_{1}\right)}u_{j+k-1}^{n-l}\right)+\mu_{2}\left(\sum_{k=0}^{j+1}w_{k}^{\left(\beta_{2}\right)}u_{j-k+1}^{n-l}+\sum_{k=0}^{M-j+1}w_{k}^{\left(\beta_{2}\right)}u_{j+k-1}^{n-l}\right)-R_{2}\right]\\ + & \left(I^{\alpha }f\left(x_{j},t_{n+1}\right)-I^{\alpha }f\left(x_{j},t_{n}\right)+R_{1}\right)\\ = & -\mu_{1}\nu^{\alpha }\left[c_{0}^{\alpha }\left(\sum_{k=0}^{j+1}w_{k}^{\left(\beta_{1}\right)}u_{j-k+1}^{n+1}+\sum_{k=0}^{M-j+1}w_{k}^{\left(\beta_{1}\right)}u_{j+k-1}^{n+1}\right)+\left(a_{n}^{\alpha }-a_{n-1}^{\alpha }\right)\left(\sum_{k=0}^{j+1}w_{k}^{\left(\beta_{1}\right)}u_{j-k+1}^{0}+\sum_{k=0}^{M-j+1}w_{k}^{\left(\beta_{1}\right)}u_{j+k-1}^{0}\right)\right.\\ & \left.+\sum_{l=0}^{n-1}\left(d_{l+1}^{\alpha }-d_{l}^{\alpha }\right)\left(\sum_{k=0}^{j+1}w_{k}^{\left(\beta_{1}\right)}u_{j-k+1}^{n-l}+\sum_{k=0}^{M-j+1}w_{k}^{\left(\beta_{1}\right)}u_{j+k-1}^{n-l}\right)\right]\\ & -\mu_{2}\nu^{\alpha }\left[c_{0}^{\alpha }\left(\sum_{k=0}^{j+1}w_{k}^{\left(\beta_{2}\right)}u_{j-k+1}^{n+1}+\sum_{k=0}^{M-j+1}w_{k}^{\left(\beta_{2}\right)}u_{j+k-1}^{n+1}\right)+\left(a_{n}^{\alpha }-a_{n-1}^{\alpha }\right)\left(\sum_{k=0}^{j+1}w_{k}^{\left(\beta_{2}\right)}u_{j-k+1}^{0}+\sum_{k=0}^{M-j+1}w_{k}^{\left(\beta_{2}\right)}u_{j+k-1}^{0}\right)\right.\\ & \left.+\sum_{l=0}^{n-1}\left(d_{l+1}^{\alpha }-d_{l}^{\alpha }\right)\left(\sum_{k=0}^{j+1}w_{k}^{\left(\beta_{2}\right)}u_{j-k+1}^{n-l}+\sum_{k=0}^{M-j+1}w_{k}^{\left(\beta_{2}\right)}u_{j+k-1}^{n-l}\right)\right]\\ & +\left(\tilde{f}\left(x_{j},t_{n+1}\right)-\tilde{f}\left(x_{j},t_{n}\right)\right)+R_{3}, \end{array}$$

where $R_{2}$ depends on *h* and by using Lemma 4, we have

$$|R_{2}|\leq Ch^{2},$$

and

$$\nu^{\alpha }=\frac{\tau^{\alpha }}{\Gamma (\alpha +1)},\;\tilde{f}\left(x_{j},t_{n}\right)=\frac{1}{\Gamma (\alpha )}\int_{0}^{t_{n}}{\left(t_{n}-s\right)}^{\alpha -1}f\left(x_{j},s\right)ds,$$

(19) $$\begin{array}{cc} R_{3} & =R_{1}+[c_{0}^{\alpha }\left(u_{j-k+1}^{n+1}+u_{j+k-1}^{n+1}\right)+\left(a_{n}^{\alpha }-a_{n-1}^{\alpha }\right)\left(u_{j-k+1}^{0}+u_{j+k-1}^{0}\right)\\ & +\sum_{l=0}^{n-1}\left(d_{l+1}^{\alpha }-d_{l}^{\alpha }\right)\left(u_{j-k+1}^{n-l}+u_{j+k-1}^{n-l}\right)]R_{2}. \end{array}$$

Here $R_{3}$ depends on τ and *h* as by Lemma 7, we get

$$\begin{array}{cc} |R_{3}|= & C\tau^{2+\alpha }b_{n}^{\alpha }+C\tau^{\alpha }h^{2}b_{n}^{\alpha }\\ = & C\tau^{\alpha }b_{n}^{\alpha }\left(\tau^{2}+h^{2}\right). \end{array}$$

Letting $\eta_{1}=\mu_{1}\nu^{\alpha }\geq 0,\;\eta_{2}=\mu_{2}\nu^{\alpha }\leq 0$, we obtain

(20) $$\begin{array}{c} u_{j}^{n+1}-u_{j}^{n}\\ =-\eta_{1}\left[c_{0}^{\alpha }\left(\sum_{k=0}^{j+1}w_{k}^{\left(\beta_{1}\right)}u_{j-k+1}^{n+1}+\sum_{k=0}^{M-j+1}w_{k}^{\left(\beta_{1}\right)}u_{j+k-1}^{n+1}\right)+\left(a_{n}^{\alpha }-a_{n-1}^{\alpha }\right)\left(\sum_{k=0}^{j+1}w_{k}^{\left(\beta_{1}\right)}u_{j-k+1}^{0}+\sum_{k=0}^{M-j+1}w_{k}^{\left(\beta_{1}\right)}u_{j+k-1}^{0}\right)\right.\\ \left.+\sum_{l=0}^{n-1}\left(d_{l+1}^{\alpha }-d_{l}^{\alpha }\right)\left(\sum_{k=0}^{j+1}w_{k}^{\left(\beta_{1}\right)}u_{j-k+1}^{n-l}+\sum_{k=0}^{M-j+1}w_{k}^{\left(\beta_{1}\right)}u_{j+k-1}^{n-l}\right)\right]\\ -\eta_{2}\left[c_{0}^{\alpha }\left(\sum_{k=0}^{j+1}w_{k}^{\left(\beta_{2}\right)}u_{j-k+1}^{n+1}+\sum_{k=0}^{M-j+1}w_{k}^{\left(\beta_{2}\right)}u_{j+k-1}^{n+1}\right)+\left(a_{n}^{\alpha }-a_{n-1}^{\alpha }\right)\left(\sum_{k=0}^{j+1}w_{k}^{\left(\beta_{2}\right)}u_{j-k+1}^{0}+\sum_{k=0}^{M-j+1}w_{k}^{\left(\beta_{2}\right)}u_{j+k-1}^{0}\right)\right.\\ \left.+\sum_{l=0}^{n-1}\left(d_{l+1}^{\alpha }-d_{l}^{\alpha }\right)\left(\sum_{k=0}^{j+1}w_{k}^{\left(\beta_{2}\right)}u_{j-k+1}^{n-l}+\sum_{k=0}^{M-j+1}w_{k}^{\left(\beta_{2}\right)}u_{j+k-1}^{n-l}\right)\right]\\ +\left(\tilde{f}\left(x_{j},t_{n+1}\right)-\tilde{f}\left(x_{j},t_{n}\right)\right)+C\tau^{\alpha }b_{n}^{\alpha }\left(\tau^{2}+h^{2}\right). \end{array}$$

Hence, the solution for system (1)–(3) can be approximated by the following scheme:

(21) $$\begin{array}{c} u_{j}^{n+1}+\eta_{1}c_{0}^{\alpha }\left(\sum_{k=0}^{j+1}w_{k}^{\left(\beta_{1}\right)}u_{j-k+1}^{n+1}+\sum_{k=0}^{M-j+1}w_{k}^{\left(\beta_{1}\right)}u_{j+k-1}^{n+1}\right)+\eta_{2}c_{0}^{\alpha }\left(\sum_{k=0}^{j+1}w_{k}^{\left(\beta_{2}\right)}u_{j-k+1}^{n+1}+\sum_{k=0}^{M-j+1}w_{k}^{\left(\beta_{2}\right)}u_{j+k-1}^{n+1}\right)\\ =u_{j}^{n}-\eta_{1}\left[\left(a_{n}^{\alpha }-a_{n-1}^{\alpha }\right)\left(\sum_{k=0}^{j+1}w_{k}^{\left(\beta_{1}\right)}u_{j-k+1}^{0}+\sum_{k=0}^{M-j+1}w_{k}^{\left(\beta_{1}\right)}u_{j+k-1}^{0}\right)+\sum_{l=0}^{n-1}\left(d_{l+1}^{\alpha }-d_{l}^{\alpha }\right)\left(\sum_{k=0}^{j+1}w_{k}^{\left(\beta_{1}\right)}u_{j-k+1}^{n-l}+\sum_{k=0}^{M-j+1}w_{k}^{\left(\beta_{1}\right)}u_{j+k-1}^{n-l}\right)\right]\\ -\eta_{2}\left[\left(a_{n}^{\alpha }-a_{n-1}^{\alpha }\right)\left(\sum_{k=0}^{j+1}w_{k}^{\left(\beta_{2}\right)}u_{j-k+1}^{0}+\sum_{k=0}^{M-j+1}w_{k}^{\left(\beta_{2}\right)}u_{j+k-1}^{0}\right)+\sum_{l=0}^{n-1}\left(d_{l+1}^{\alpha }-d_{l}^{\alpha }\right)\left(\sum_{k=0}^{j+1}w_{k}^{\left(\beta_{2}\right)}u_{j-k+1}^{n-l}+\sum_{k=0}^{M-j+1}w_{k}^{\left(\beta_{2}\right)}u_{j+k-1}^{n-l}\right)\right]\\ +({\tilde{f}}_{j}^{n+1}-{\tilde{f}}_{j}^{n}), \end{array}$$

where ${\tilde{f}}_{j}^{n}=\tilde{f}\left(x_{j},t_{n}\right)$. We can write this system into the following matrix form:

(22) $$\begin{array}{c} \left(I+\eta_{1}c_{0}^{\alpha }\left(B_{1}+B_{1}^{T}\right)+c_{0}^{\alpha }\eta_{2}\left(B_{2}+B_{2}^{T}\right)\right)U^{n+1}\\ =U^{n}-\left(a_{n}^{\alpha }-a_{n-1}^{\alpha }\right)\left(\eta_{1}\left(B_{1}+B_{1}^{T}\right)+\eta_{2}\left(B_{2}+B_{2}^{T}\right)\right)U^{0}-\left(\eta_{1}\left(B_{1}+B_{1}^{T}\right)+\eta_{2}\left(B_{2}+B_{2}^{T}\right)\right)\sum_{l=0}^{n-1}\left(d_{l+1}^{\alpha }-d_{l}^{\alpha }\right)U^{n-l}\\ +\left({\tilde{F}}^{n+1}-{\tilde{F}}^{n}\right)+C\tau^{\alpha }b_{n}^{\alpha }\left(\tau^{2}+h^{2}\right), \end{array}$$

where

$$U^{n}={\left(u_{1}^{n},u_{2}^{n},\ldots ,u_{M-1}^{n}\right)}^{T},\;\;{\tilde{F}}^{n}={\left({\tilde{f}}_{1}^{n},{\tilde{f}}_{2}^{n},\ldots ,{\tilde{f}}_{M-1}^{n}\right)}^{T},$$

with *I* is an (M−1)×(M−1) identity matrix, $B_{1}$ and $B_{2}$ are (M−1)×(M−1) matrices that satisfy

(23) $$B_{1}=\left(\begin{array}{cccccc} w_{1}^{\left(\beta_{1}\right)} & w_{0}^{\left(\beta_{1}\right)} & 0 & 0 & \ldots & 0\\ w_{2}^{\left(\beta_{1}\right)} & w_{1}^{\left(\beta_{1}\right)} & w_{0}^{\beta_{2}} & 0 & \ldots & 0\\ w_{3}^{\left(\beta_{1}\right)} & w_{2}^{\left(\beta_{1}\right)} & w_{1}^{\beta_{2}} & 0 & \ldots & 0\\ \ldots & \ldots & \ldots & \ldots & \ldots & \ldots \\ w_{M-2}^{\left(\beta_{1}\right)} & w_{M-3}^{\left(\beta_{1}\right)} & w_{M-4}^{\left(\beta_{1}\right)} & \ldots & w_{1}^{\left(\beta_{1}\right)} & w_{0}^{\left(\beta_{1}\right)}\\ w_{M-1}^{\left(\beta_{1}\right)} & w_{M-2}^{\left(\beta_{1}\right)} & w_{M-3}^{\left(\beta_{1}\right)} & \ldots & w_{2}^{\left(\beta_{1}\right)} & w_{1}^{\left(\beta_{1}\right)} \end{array}\right),$$

(24) $$B_{2}=\left(\begin{array}{cccccc} w_{1}^{\left(\beta_{2}\right)} & w_{0}^{\left(\beta_{2}\right)} & 0 & 0 & \ldots & 0\\ w_{2}^{\left(\beta_{2}\right)} & w_{1}^{\left(\beta_{2}\right)} & w_{0}^{\left(\beta_{2}\right)} & 0 & \ldots & 0\\ w_{3}^{\left(\beta_{2}\right)} & w_{2}^{\left(\beta_{2}\right)} & w_{1}^{\left(\beta_{2}\right)} & 0 & \ldots & 0\\ \ldots & \ldots & \ldots & \ldots & \ldots & \ldots \\ w_{M-2}^{\left(\beta_{2}\right)} & w_{M-3}^{\left(\beta_{2}\right)} & w_{M-4}^{\left(\beta_{2}\right)} & \ldots & w_{1}^{\left(\beta_{2}\right)} & w_{0}^{\left(\beta_{2}\right)}\\ w_{M-1}^{\left(\beta_{2}\right)} & w_{M-2}^{\left(\beta_{2}\right)} & w_{M-3}^{\left(\beta_{2}\right)} & \ldots & w_{2}^{\left(\beta_{2}\right)} & w_{1}^{\left(\beta_{2}\right)} \end{array}\right).$$

Let

(25) $$B_{1}=B_{1}+B_{1}^{T},\;B_{2}=B_{2}+B_{2}^{T},\;A=\eta_{1}B_{1}+\eta_{2}B_{2},$$

then we obtain the following numerical scheme based on fractional trapezoid formula

(26) $$\left(I+c_{0}^{\alpha }A\right)U^{n+1}=U^{n}-A\left(a_{n}^{\alpha }-a_{n-1}^{\alpha }\right)U^{0}-A\sum_{l=0}^{n-1}\left(d_{l+1}^{\alpha }-d_{l}^{\alpha }\right)U^{n-l}+\left({\tilde{F}}^{n+1}-{\tilde{F}}^{n}\right),$$

with initial and boundary conditions

(27) $$\left\{\begin{array}{cc} U\left(0\right)=U^{0} & \\ u_{0}^{n}=u_{M}^{n}=0,\;\;\;\;1\leq n\leq N. & \end{array}\right.$$

## 4. Stability and Convergence Analysis

### 4.1. Stability

In this section, we analyze the stability and convergence for the scheme (26).

**Remark** **1.** *For $n=0,\;d_{0}^{\alpha }=c_{0}^{\alpha }=\frac{1}{1+\alpha },\;d_{1}^{\alpha }=a_{0}^{\alpha }+c_{1}^{\alpha }=\frac{2^{\alpha +1}-2}{\alpha +1}.$ If*

(28) $$2^{\alpha +1}\leq 3,$$

*then we obtain*

$$d_{0}^{\alpha }>d_{1}^{\alpha }.$$

**Remark** **2.** *Let $b_{n}^{\alpha }$ be as defined in Lemma 8, then $c_{0}^{\alpha }\geq b_{n}^{\alpha },\;\;\;n=0,1,2,\ldots ,$* *where $0<c_{0}^{\alpha }=\frac{1}{1+\alpha }<1,\;0<b_{n}^{\alpha }<1,\;\;\;n=0,1,2,\ldots .$ Using $d_{n}^{\alpha }=a_{n-1}^{\alpha }+c_{n}^{\alpha }$, we have*

$$\begin{array}{cc} 0< & a_{n-1}^{\alpha }-a_{n}^{\alpha }+d_{0}^{\alpha }-d_{n}^{\alpha }\\ = & a_{n-1}^{\alpha }-a_{n}^{\alpha }+d_{0}^{\alpha }-\left(a_{n-1}^{\alpha }+c_{n}^{\alpha }\right)\\ = & d_{0}^{\alpha }-\left(a_{n}^{\alpha }+c_{n}^{\alpha }\right)=d_{0}^{\alpha }-b_{n}^{\alpha }=c_{0}^{\alpha }-b_{n}^{\alpha }. \end{array}$$

**Lemma** **11.** *$w_{2}^{\left(\beta_{2}\right)}\geq 0$ if*

(29) $$\frac{\sqrt{17}-1}{2}\leq \beta_{2}\leq 2.$$

**Proof.**  Since $w_{2}^{\left(\beta_{2}\right)}\geq 0$ is equivalent to

$$\beta_{2}^{2}+\beta_{2}-4\geq 0,$$

that is

$$\frac{\sqrt{17}-1}{2}\leq \beta_{2}\leq 2.$$

 ☐

Let ${\tilde{u}}_{j}^{n}$ numerical solution of the numerical scheme (21) with a different initial value ${\tilde{u}}_{j}^{0}$, and

$$\varepsilon_{j}^{n}={\tilde{u}}_{j}^{n}-u_{j}^{n},\;n=0,1,\ldots N,j=1,2,\ldots M-1.$$

According to (21), we have

(30) $$\begin{array}{c} \varepsilon_{j}^{n+1}+c_{0}^{\alpha }\left[\eta_{1}\left(\sum_{k=0}^{j+1}w_{k}^{\left(\beta_{1}\right)}\varepsilon_{j-k+1}^{n+1}+\sum_{k=0}^{M-j+1}w_{k}^{\left(\beta_{1}\right)}\varepsilon_{j+k-1}^{n+1}\right)+\eta_{2}\left(\sum_{k=0}^{j+1}w_{k}^{\left(\beta_{2}\right)}\varepsilon_{j-k+1}^{n+1}+\sum_{k=0}^{M-j+1}w_{k}^{\left(\beta_{2}\right)}\varepsilon_{j+k-1}^{n+1}\right)\right]\\ =\varepsilon_{j}^{n}-\left(a_{n}^{\alpha }-a_{n-1}^{\alpha }\right)\left[\eta_{1}\left(\sum_{k=0}^{j+1}w_{k}^{\left(\beta_{1}\right)}\varepsilon_{j-k+1}^{0}+\sum_{k=0}^{M-j+1}w_{k}^{\left(\beta_{1}\right)}\varepsilon_{j+k-1}^{0}\right)+\eta_{2}\left(\sum_{k=0}^{j+1}w_{k}^{\left(\beta_{2}\right)}\varepsilon_{j-k+1}^{0}+\sum_{k=0}^{M-j+1}w_{k}^{\left(\beta_{2}\right)}\varepsilon_{j+k-1}^{0}\right)\right]\\ -\sum_{l=0}^{n-1}\left(d_{l+1}^{\alpha }-d_{l}^{\alpha }\right)\left[\eta_{1}\left(\sum_{k=0}^{j+1}w_{k}^{\left(\beta_{1}\right)}\varepsilon_{j-k+1}^{n-l}+\sum_{k=0}^{M-j+1}w_{k}^{\left(\beta_{1}\right)}\varepsilon_{j+k-1}^{n-l}\right)+\eta_{2}\left(\sum_{k=0}^{j+1}w_{k}^{\left(\beta_{2}\right)}\varepsilon_{j-k+1}^{n-l}+\sum_{k=0}^{M-j+1}w_{k}^{\left(\beta_{2}\right)}\varepsilon_{j+k-1}^{n-l}\right)\right]. \end{array}$$

For convenience, we suppose that

$$S^{\left(\beta \right)}=\sum_{k=0}^{j+1}w_{k}^{\left(\beta \right)},\;{\bar{S}}^{\left(\beta \right)}=\sum_{k=0}^{M-j+1}w_{k}^{\left(\beta \right)}.$$

Put $E^{n}=\left(\varepsilon_{1}^{n},\varepsilon_{2}^{n},\ldots ,\varepsilon_{M-1}^{n}\right),\;n=0,1,2,\ldots N$, and assume that

$$∥E^{n}{∥}_{\infty }=\max_{1\leq j\leq M-1}|\varepsilon_{j}^{n}\left|=\right|\varepsilon_{\hat{j}}^{n}|.$$

**Theorem** **1.** *Suppose (28) and (29) hold. Then, the fractional numerical scheme (26) is stable, i.e.,*

$$∥E^{n+1}{∥}_{\infty }\leq {∥E^{0}∥}_{\infty },\;\;\;\;n=0,1,\ldots ,N-1.$$

**Proof.**  We prove by mathematical induction. For n=0, (30) can be written as

(31) $$\begin{array}{c} \varepsilon_{j}^{1}+c_{0}^{\alpha }\left[\eta_{1}\left(\sum_{k=0}^{j+1}w_{k}^{\left(\beta_{1}\right)}\varepsilon_{j-k+1}^{1}+\sum_{k=0}^{M-j+1}w_{k}^{\left(\beta_{1}\right)}\varepsilon_{j+k-1}^{1}\right)+\eta_{2}\left(\sum_{k=0}^{j+1}w_{k}^{\left(\beta_{2}\right)}\varepsilon_{j-k+1}^{1}+\sum_{k=0}^{M-j+1}w_{k}^{\left(\beta_{2}\right)}\varepsilon_{j+k-1}^{1}\right)\right]\\ =\varepsilon_{j}^{0}-a_{1}^{\alpha }\left[\eta_{1}\left(\sum_{k=0}^{j+1}w_{k}^{\left(\beta_{1}\right)}\varepsilon_{j-k+1}^{0}+\sum_{k=0}^{M-j+1}w_{k}^{\left(\beta_{1}\right)}\varepsilon_{j+k-1}^{0}\right)+\eta_{2}\left(\sum_{k=0}^{j+1}w_{k}^{\left(\beta_{2}\right)}\varepsilon_{j-k+1}^{0}+\sum_{k=0}^{M-j+1}w_{k}^{\left(\beta_{2}\right)}\varepsilon_{j+k-1}^{0}\right)\right], \end{array}$$

that is

(32) $$\begin{array}{c} \varepsilon_{j}^{1}+c_{0}^{\alpha }\left[\eta_{1}\left(\sum_{k=0}^{j+1}w_{k}^{\left(\beta_{1}\right)}\varepsilon_{j-k+1}^{1}+\sum_{k=0}^{M-j+1}w_{k}^{\left(\beta_{1}\right)}\varepsilon_{j+k-1}^{1}\right)+\eta_{2}\left(\sum_{k=0}^{j+1}w_{k}^{\left(\beta_{2}\right)}\varepsilon_{j-k+1}^{1}+\sum_{k=0}^{M-j+1}w_{k}^{\left(\beta_{2}\right)}\varepsilon_{j+k-1}^{1}\right)\right]\\ +a_{1}^{\alpha }\left[\eta_{1}\left(\sum_{k=0}^{j+1}w_{k}^{\left(\beta_{1}\right)}\varepsilon_{j-k+1}^{0}+\sum_{k=0}^{M-j+1}w_{k}^{\left(\beta_{1}\right)}\varepsilon_{j+k-1}^{0}\right)+\eta_{2}\left(\sum_{k=0}^{j+1}w_{k}^{\left(\beta_{2}\right)}\varepsilon_{j-k+1}^{0}+\sum_{k=0}^{M-j+1}w_{k}^{\left(\beta_{2}\right)}\varepsilon_{j+k-1}^{0}\right)\right]\\ =\varepsilon_{j}^{0}. \end{array}$$

Using $|v_{1}\left|-\right|v_{2}\left|\leq \right|v_{1}-v_{2}|$ and (32), we have

$$\begin{array}{cc} & ∥E^{1}{∥}_{\infty }=\left|\varepsilon_{\hat{j}}^{1}\right|\\ \leq & |\varepsilon_{\hat{j}}^{1}|+c_{0}^{\alpha }\left[\eta_{1}\left(\sum_{k=0}^{j+1}w_{k}^{\left(\beta_{1}\right)}|\varepsilon_{\hat{j}}^{1}|+\sum_{k=0}^{M-j+1}w_{k}^{\left(\beta_{1}\right)}\left|\varepsilon_{\hat{j}}^{1}\right|\right)+\eta_{2}\left(\sum_{k=0}^{j+1}w_{k}^{\left(\beta_{2}\right)}|\varepsilon_{\hat{j}}^{1}|+\sum_{k=0}^{M-j+1}w_{k}^{\left(\beta_{2}\right)}\left|\varepsilon_{\hat{j}}^{1}\right|\right)\right]\\ & +a_{0}^{\alpha }\left[\eta_{1}\left(\sum_{k=0}^{j+1}w_{k}^{\left(\beta_{1}\right)}|\varepsilon_{\hat{j}}^{0}|+\sum_{k=0}^{M-j+1}w_{k}^{\left(\beta_{1}\right)}\left|\varepsilon_{\hat{j}}^{0}\right|\right)+\eta_{2}\left(\sum_{k=0}^{j+1}w_{k}^{\left(\beta_{2}\right)}|\varepsilon_{\hat{j}}^{0}|+\sum_{k=0}^{M-j+1}w_{k}^{\left(\beta_{2}\right)}\left|\varepsilon_{\hat{j}}^{0}\right|\right)\right]\\ \leq & |\varepsilon_{\hat{j}}^{1}|+c_{0}^{\alpha }\left[\eta_{1}\left(\sum_{k=0}^{j+1}w_{k}^{\left(\beta_{1}\right)}|\varepsilon_{\hat{j}-k+1}^{1}|+\sum_{k=0}^{M-j+1}w_{k}^{\left(\beta_{1}\right)}\left|\varepsilon_{\hat{j}+k-1}^{1}\right|\right)+\eta_{2}\left(\sum_{k=0}^{j+1}w_{k}^{\left(\beta_{2}\right)}|\varepsilon_{\hat{j}-k+1}^{1}|+\sum_{k=0}^{M-j+1}w_{k}^{\left(\beta_{2}\right)}\left|\varepsilon_{\hat{j}+k-1}^{1}\right|\right)\right]\\ & +a_{0}^{\alpha }\left[\eta_{1}\left(\sum_{k=0}^{j+1}w_{k}^{\left(\beta_{1}\right)}|\varepsilon_{\hat{j}-k+1}^{0}|+\sum_{k=0}^{M-j+1}w_{k}^{\left(\beta_{1}\right)}\left|\varepsilon_{\hat{j}+k-1}^{0}\right|\right)+\eta_{2}\left(\sum_{k=0}^{j+1}w_{k}^{\left(\beta_{2}\right)}|\varepsilon_{\hat{j}-k+1}^{0}|+\sum_{k=0}^{M-j+1}w_{k}^{\left(\beta_{2}\right)}\left|\varepsilon_{\hat{j}+k-1}^{0}\right|\right)\right]\\ = & \left(\right|\varepsilon_{\hat{j}}^{1}|+2(c_{0}^{\alpha }|\varepsilon_{\hat{j}}^{1}|+a_{0}^{\alpha }|\varepsilon_{\hat{j}}^{0}\left|\right)\left(\eta_{1}w_{1}^{\left(\beta_{1}\right)}+\eta_{2}w_{1}^{\left(\beta_{2}\right)}\right))\\ & +c_{0}^{\alpha }\left[\eta_{1}\left(\sum_{k=0,k\neq 1}^{j+1}w_{k}^{\left(\beta_{1}\right)}|\varepsilon_{\hat{j}-k+1}^{1}|+\sum_{k=0,k\neq 1}^{M-j+1}w_{k}^{\left(\beta_{1}\right)}\left|\varepsilon_{\hat{j}+k-1}^{1}\right|\right)+\eta_{2}\left(\sum_{k=0,k\neq 1}^{j+1}w_{k}^{\left(\beta_{2}\right)}|\varepsilon_{\hat{j}-k+1}^{1}|+\sum_{k=0,k\neq 1}^{M-j+1}w_{k}^{\left(\beta_{2}\right)}\left|\varepsilon_{\hat{j}+k-1}^{1}\right|\right)\right]\\ & +a_{0}^{\alpha }\left[\eta_{1}\left(\sum_{k=0,k\neq 1}^{j+1}w_{k}^{\left(\beta_{1}\right)}|\varepsilon_{\hat{j}-k+1}^{0}|+\sum_{k=0,k\neq 1}^{M-j+1}w_{k}^{\left(\beta_{1}\right)}\left|\varepsilon_{\hat{j}+k-1}^{0}\right|\right)+\eta_{2}\left(\sum_{k=0,k\neq 1}^{j+1}w_{k}^{\left(\beta_{2}\right)}|\varepsilon_{\hat{j}-k+1}^{0}|+\sum_{k=0,k\neq 1}^{M-j+1}w_{k}^{\left(\beta_{2}\right)}\left|\varepsilon_{\hat{j}+k-1}^{0}\right|\right)\right]\\ \leq & \left|\varepsilon_{\hat{j}}^{1}+2(c_{0}^{\alpha }|\varepsilon_{\hat{j}}^{1}|+a_{0}^{\alpha }|\varepsilon_{\hat{j}}^{0}\left|\right)\left(\eta_{1}w_{1}^{\left(\beta_{1}\right)}+\eta_{2}w_{1}^{\left(\beta_{2}\right)}\right)\right.\\ & +c_{0}^{\alpha }\left[\eta_{1}\left(\sum_{k=0,k\neq 1}^{j+1}w_{k}^{\left(\beta_{1}\right)}\varepsilon_{\hat{j}-k+1}^{1}+\sum_{k=0,k\neq 1}^{M-j+1}w_{k}^{\left(\beta_{1}\right)}\varepsilon_{\hat{j}+k-1}^{1}\right)+\eta_{2}\left(\sum_{k=0,k\neq 1}^{j+1}w_{k}^{\left(\beta_{2}\right)}\varepsilon_{\hat{j}-k+1}^{1}+\sum_{k=0,k\neq 1}^{M-j+1}w_{k}^{\left(\beta_{2}\right)}\varepsilon_{\hat{j}+k-1}^{1}\right)\right]\\ & \left.+a_{0}^{\alpha }\left[\eta_{1}\left(\sum_{k=0,k\neq 1}^{j+1}w_{k}^{\left(\beta_{1}\right)}|\varepsilon_{\hat{j}-k+1}^{0}|+\sum_{k=0,k\neq 1}^{M-j+1}w_{k}^{\left(\beta_{1}\right)}\left|\varepsilon_{\hat{j}+k-1}^{0}\right|\right)+\eta_{2}\left(\sum_{k=0,k\neq 1}^{j+1}w_{k}^{\left(\beta_{2}\right)}|\varepsilon_{\hat{j}-k+1}^{0}|+\sum_{k=0,k\neq 1}^{M-j+1}w_{k}^{\left(\beta_{2}\right)}\left|\varepsilon_{\hat{j}+k-1}^{0}\right|\right)\right]\right|\\ = & \left|\varepsilon_{\hat{j}}^{1}+c_{0}^{\alpha }\left[\eta_{1}\left(\sum_{k=0}^{j+1}w_{k}^{\left(\beta_{1}\right)}\varepsilon_{\hat{j}-k+1}^{1}+\sum_{k=0}^{M-j+1}w_{k}^{\left(\beta_{1}\right)}\varepsilon_{\hat{j}+k-1}^{1}\right)+\eta_{2}\left(\sum_{k=0}^{j+1}w_{k}^{\left(\beta_{2}\right)}\varepsilon_{\hat{j}-k+1}^{1}+\sum_{k=0}^{M-j+1}w_{k}^{\left(\beta_{2}\right)}\varepsilon_{\hat{j}+k-1}^{1}\right)\right]\right.\\ & \left.+a_{0}^{\alpha }\left[\eta_{1}\left(\sum_{k=0}^{j+1}w_{k}^{\left(\beta_{1}\right)}\varepsilon_{\hat{j}-k+1}^{0}+\sum_{k=0}^{M-j+1}w_{k}^{\left(\beta_{1}\right)}\varepsilon_{\hat{j}+k-1}^{0}\right)+\eta_{2}\left(\sum_{k=0}^{j+1}w_{k}^{\left(\beta_{2}\right)}\varepsilon_{\hat{j}-k+1}^{0}+\sum_{k=0}^{M-j+1}w_{k}^{\left(\beta_{2}\right)}\varepsilon_{\hat{j}+k-1}^{0}\right)\right]\right|\\ = & |\varepsilon_{\hat{j}}^{0}|. \end{array}$$

Hence, we obtain

$$∥E^{1}{∥}_{\infty }\leq {∥E^{0}∥}_{\infty }.$$

Now suppose that

$$∥E^{k}{∥}_{\infty }\leq {∥E^{0}∥}_{\infty },\;k=0,1,2,\ldots ,n.$$

Using $|v_{1}\left|-\right|v_{2}\left|\leq \right|v_{1}-v_{1}|$ and (30), we have

$$\begin{array}{cc} & \left(1+c_{0}^{\alpha }\left[\eta_{1}\left(\sum_{k=0}^{j+1}w_{k}^{\left(\beta_{1}\right)}+\sum_{k=0}^{M-j+1}w_{k}^{\left(\beta_{1}\right)}\right)+\eta_{2}\left(\sum_{k=0}^{j+1}w_{k}^{\left(\beta_{2}\right)}+\sum_{k=0}^{M-j+1}w_{k}^{\left(\beta_{2}\right)}\right)\right]\right)\left|\varepsilon_{\hat{j}}^{n+1}\right|\\ \leq & |\varepsilon_{\hat{j}}^{n+1}|+c_{0}^{\alpha }\left[\eta_{1}\left(\sum_{k=0}^{j+1}w_{k}^{\left(\beta_{1}\right)}|\varepsilon_{\hat{j}-k+1}^{n+1}|+\sum_{k=0}^{M-j+1}w_{k}^{\left(\beta_{1}\right)}\left|\varepsilon_{\hat{j}+k-1}^{n+1}\right|\right)+\eta_{2}\left(\sum_{k=0}^{j+1}w_{k}^{\left(\beta_{2}\right)}|\varepsilon_{\hat{j}-k+1}^{n+1}|+\sum_{k=0}^{M-j+1}w_{k}^{\left(\beta_{2}\right)}\left|\varepsilon_{\hat{j}+k-1}^{n+1}\right|\right)\right]\\ = & \left(1+2c_{0}^{\alpha }\left(\eta_{1}w_{0}^{\left(\beta_{1}\right)}+\eta_{2}w_{1}^{\left(\beta_{2}\right)}\right)|\varepsilon_{\hat{j}}^{n+1}|\right)\\ & +c_{0}^{\alpha }\left[\eta_{1}\left(\sum_{k=0,k\neq 1}^{j+1}w_{k}^{\left(\beta_{1}\right)}|\varepsilon_{\hat{j}-k+1}^{n+1}|+\sum_{k=0,k\neq 1}^{M-j+1}w_{k}^{\left(\beta_{1}\right)}\left|\varepsilon_{\hat{j}+k-1}^{n+1}\right|\right)+\eta_{2}\left(\sum_{k=0,k\neq 1}^{j+1}w_{k}^{\left(\beta_{2}\right)}|\varepsilon_{\hat{j}-k+1}^{n+1}|+\sum_{k=0,k\neq 1}^{M-j+1}w_{k}^{\left(\beta_{2}\right)}\left|\varepsilon_{\hat{j}+k-1}^{n+1}\right|\right)\right]\\ \leq & \left|\varepsilon_{\hat{j}}^{n+1}+c_{0}^{\alpha }\left[\eta_{1}\left(\sum_{k=0}^{j+1}w_{k}^{\left(\beta_{1}\right)}\varepsilon_{\hat{j}-k+1}^{n+1}+\sum_{k=0}^{M-j+1}w_{k}^{\left(\beta_{1}\right)}\varepsilon_{\hat{j}+k-1}^{n+1}\right)+\eta_{2}\left(\sum_{k=0}^{j+1}w_{k}^{\left(\beta_{2}\right)}\varepsilon_{\hat{j}-k+1}^{n+1}+\sum_{k=0}^{M-j+1}w_{k}^{\left(\beta_{2}\right)}\varepsilon_{\hat{j}+k-1}^{n+1}\right)\right]\right|\\ = & \left|\varepsilon_{\hat{j}}^{n}-\left(a_{n}^{\alpha }-a_{n-1}^{\alpha }\right)\left[\eta_{1}\left(\sum_{k=0}^{j+1}w_{k}^{\left(\beta_{1}\right)}\varepsilon_{\hat{j}-k+1}^{0}+\sum_{k=0}^{M-j+1}w_{k}^{\left(\beta_{1}\right)}\varepsilon_{\hat{j}+k-1}^{0}\right)+\eta_{2}\left(\sum_{k=0}^{j+1}w_{k}^{\left(\beta_{2}\right)}\varepsilon_{\hat{j}-k+1}^{0}+\sum_{k=0}^{M-j+1}w_{k}^{\left(\beta_{2}\right)}\varepsilon_{\hat{j}+k-1}^{0}\right)\right]\right.\\ & \left.-\sum_{l=0}^{n-1}\left(d_{l+1}^{\alpha }-d_{l}^{\alpha }\right)\left[\eta_{1}\left(\sum_{k=0}^{j+1}w_{k}^{\left(\beta_{1}\right)}\varepsilon_{\hat{j}-k+1}^{n-l}+\sum_{k=0}^{M-j+1}w_{k}^{\left(\beta_{1}\right)}\varepsilon_{\hat{j}+k-1}^{n-l}\right)+\eta_{2}\left(\sum_{k=0}^{j+1}w_{k}^{\left(\beta_{2}\right)}\varepsilon_{\hat{j}-k+1}^{n-l}+\sum_{k=0}^{M-j+1}w_{k}^{\left(\beta_{2}\right)}\varepsilon_{\hat{j}+k-1}^{n-l}\right)\right]\right|\\ \leq & |\varepsilon_{\hat{j}}^{n}|-\left(a_{n}^{\alpha }-a_{n-1}^{\alpha }\right)\left[\eta_{1}\left(\sum_{k=0}^{j+1}w_{k}^{\left(\beta_{1}\right)}|\varepsilon_{\hat{j}-k+1}^{0}|+\sum_{k=0}^{M-j+1}w_{k}^{\left(\beta_{1}\right)}\left|\varepsilon_{\hat{j}+k-1}^{0}\right|\right)+\eta_{2}\left(\sum_{k=0}^{j+1}w_{k}^{\left(\beta_{2}\right)}|\varepsilon_{\hat{j}-k+1}^{0}|+\sum_{k=0}^{M-j+1}w_{k}^{\left(\beta_{2}\right)}\left|\varepsilon_{\hat{j}+k-1}^{0}\right|\right)\right]\\ & -\sum_{l=0}^{n-1}\left(d_{l+1}^{\alpha }-d_{l}^{\alpha }\right)\left[\eta_{1}\left(\sum_{k=0}^{j+1}w_{k}^{\left(\beta_{1}\right)}|\varepsilon_{\hat{j}-k+1}^{n-1}|+\sum_{k=0}^{M-j+1}w_{k}^{\left(\beta_{1}\right)}\left|\varepsilon_{\hat{j}+k-1}^{n-1}\right|\right)+\eta_{2}\left(\sum_{k=0}^{j+1}w_{k}^{\left(\beta_{2}\right)}|\varepsilon_{\hat{j}-k+1}^{n-1}|+\sum_{k=0}^{M-j+1}w_{k}^{\left(\beta_{2}\right)}\left|\varepsilon_{\hat{j}+k-1}^{n-1}\right|\right)\right]\\ = & ∥E^{0}∥-\left(a_{n}^{\alpha }-a_{n-1}^{\alpha }\right)\left[\eta_{1}\left(\sum_{k=0}^{j+1}w_{k}^{\left(\beta_{1}\right)}∥E^{0}∥+\sum_{k=0}^{M-j+1}w_{k}^{\left(\beta_{1}\right)}∥E^{0}∥\right)+\eta_{2}\left(\sum_{k=0}^{j+1}w_{k}^{\left(\beta_{2}\right)}∥E^{0}∥+\sum_{k=0}^{M-j+1}w_{k}^{\left(\beta_{2}\right)}∥E^{0}∥\right)\right]\\ & -\left(d_{n}^{\alpha }-d_{0}^{\alpha }\right)\left[\eta_{1}\left(\sum_{k=0}^{j+1}w_{k}^{\left(\beta_{1}\right)}∥E^{0}∥+\sum_{k=0}^{M-j+1}w_{k}^{\left(\beta_{1}\right)}∥E^{0}∥\right)+\eta_{2}\left(\sum_{k=0}^{j+1}w_{k}^{\left(\beta_{2}\right)}∥E^{0}∥+\sum_{k=0}^{M-j+1}w_{k}^{\left(\beta_{2}\right)}∥E^{0}∥\right)\right]\\ = & \left(1-\left(a_{n}^{\alpha }-a_{n-1}^{\alpha }+d_{n}^{\alpha }-d_{0}^{\alpha }\right)\left[\eta_{1}\left(\sum_{k=0}^{j+1}w_{k}^{\left(\beta_{1}\right)}+\sum_{k=0}^{M-j+1}w_{k}^{\left(\beta_{1}\right)}\right)+\eta_{2}\left(\sum_{k=0}^{j+1}w_{k}^{\left(\beta_{2}\right)}+\sum_{k=0}^{M-j+1}w_{k}^{\left(\beta_{2}\right)}\right)\right]\right)∥E^{0}∥\\ = & \left(1+\left(c_{0}^{\alpha }-b_{n}^{\alpha }\right)\left[\eta_{1}\left(\sum_{k=0}^{j+1}w_{k}^{\left(\beta_{1}\right)}+\sum_{k=0}^{M-j+1}w_{k}^{\left(\beta_{1}\right)}\right)+\eta_{2}\left(\sum_{k=0}^{j+1}w_{k}^{\left(\beta_{2}\right)}+\sum_{k=0}^{M-j+1}w_{k}^{\left(\beta_{2}\right)}\right)\right]\right)∥E^{0}∥, \end{array}$$

hence

$$\begin{array}{cc} ∥E^{n+1}{∥}_{\infty } & \leq \frac{1+\left(c_{0}^{\alpha }-b_{n}^{\alpha }\right)\left[\eta_{1}\left(S^{\left(\beta_{1}\right)}+{\bar{S}}^{\left(\beta_{1}\right)}\right)+\eta_{2}\left(S^{\left(\beta_{2}\right)}+{\bar{S}}^{\left(\beta_{2}\right)}\right)\right]}{1+c_{0}^{\alpha }\left[\eta_{1}\left(S^{\left(\beta_{1}\right)}+{\bar{S}}^{\left(\beta_{1}\right)}\right)+\eta_{2}\left(S^{\left(\beta_{2}\right)}+{\bar{S}}^{\left(\beta_{2}\right)}\right)\right]}{∥E^{0}∥}_{\infty }\\ & \leq ∥E^{0}{∥}_{\infty }. \end{array}$$

 ☐

### 4.2. Convergence

Let the error at the grid points $\left(x_{j},t_{n}\right)$ be defined by

$$e_{j}^{n}=u\left(x_{j},t_{n}\right)-u_{j}^{n},\;n=0,1,\ldots N,j=1,2,\ldots M-1,$$

and we denote $E^{n}={\left(e_{1}^{n},e_{2}^{n},\ldots ,e_{M-1}^{n}\right)}^{T}.$ According to (22), the error satisfies

(33) $$\begin{array}{c} e_{j}^{n+1}+c_{0}^{\alpha }\left[\eta_{1}\left(\sum_{k=0}^{j+1}w_{k}^{\left(\beta_{1}\right)}e_{j-k+1}^{n+1}+\sum_{k=0}^{M-j+1}w_{k}^{\left(\beta_{1}\right)}e_{j+k-1}^{n+1}\right)+\eta_{2}\left(\sum_{k=0}^{j+1}w_{k}^{\left(\beta_{2}\right)}e_{j-k+1}^{n+1}+\sum_{k=0}^{M-j+1}w_{k}^{\left(\beta_{2}\right)}e_{j+k-1}^{n+1}\right)\right]\\ =e_{j}^{n}-\sum_{l=0}^{n-1}\left(d_{l+1}^{\alpha }-d_{l}^{\alpha }\right)\left[\eta_{1}\left(\sum_{k=0}^{j+1}w_{k}^{\left(\beta_{1}\right)}e_{j-k+1}^{n-l}+\sum_{k=0}^{M-j+1}w_{k}^{\left(\beta_{1}\right)}e_{j+k-1}^{n-l}\right)+\eta_{2}\left(\sum_{k=0}^{j+1}w_{k}^{\left(\beta_{2}\right)}e_{j-k+1}^{n-l}+\sum_{k=0}^{M-j+1}w_{k}^{\left(\beta_{2}\right)}e_{j+k-1}^{n-l}\right)\right]\\ +\tau^{\alpha }b_{n}^{\alpha }C\left(\tau^{2}+h^{2}\right). \end{array}$$

Put $E^{n}=\left(e_{1}^{n},e_{2}^{n},\ldots ,e_{M-1}^{n}\right),\;n=0,1,2,\ldots N$, and assume that

$$∥E^{n}{∥}_{\infty }=\max_{1\leq j\leq M-1}|e_{j}^{n}\left|=\right|e_{\hat{j}}^{n}|.$$

**Theorem** **2.** *Suppose (28) and (29) hold. Then, the fractional numerical scheme (26) is convergent with accuracy $O(\tau^{2}+h^{2})$, that is, there exists a positive constant C such that*

$$∥E^{n+1}{∥}_{\infty }\leq C\left(\tau^{2}+h^{2}\right),\;\;\;\;n=0,1,\ldots ,N-1.$$

**Proof.**  We prove by mathematical induction. For n=0, (33) can be written as

(34) $$\begin{array}{c} e_{j}^{1}+c_{0}^{\alpha }\left[\eta_{1}\left(\sum_{k=0}^{j+1}w_{k}^{\left(\beta_{1}\right)}e_{j-k+1}^{1}+\sum_{k=0}^{M-j+1}w_{k}^{\left(\beta_{1}\right)}e_{j+k-1}^{1}\right)+\eta_{2}\left(\sum_{k=0}^{j+1}w_{k}^{\left(\beta_{2}\right)}e_{j-k+1}^{1}+\sum_{k=0}^{M-j+1}w_{k}^{\left(\beta_{2}\right)}e_{j+k-1}^{1}\right)\right]\\ =\tau^{\alpha }b_{0}^{\alpha }C\left(\tau^{2}+h^{2}\right). \end{array}$$

Using $|v_{1}\left|-\right|v_{2}\left|\leq \right|v_{1}-v_{2}|$ and (34), we have

$$\begin{array}{cc} & ∥E^{1}{∥}_{\infty }=\left|e_{\hat{j}}^{1}\right|\\ \leq & |e_{\hat{j}}^{1}|+c_{0}^{\alpha }\left[\eta_{1}\left(\sum_{k=0}^{j+1}w_{k}^{\left(\beta_{1}\right)}|e_{\hat{j}}^{1}|+\sum_{k=0}^{M-j+1}w_{k}^{\left(\beta_{1}\right)}\left|e_{\hat{j}}^{1}\right|\right)+\eta_{2}\left(\sum_{k=0}^{j+1}w_{k}^{\left(\beta_{2}\right)}|e_{\hat{j}}^{1}|+\sum_{k=0}^{M-j+1}w_{k}^{\left(\beta_{2}\right)}\left|e_{\hat{j}}^{1}\right|\right)\right]\\ \leq & |e_{\hat{j}}^{1}|+c_{0}^{\alpha }\left[\eta_{1}\left(\sum_{k=0}^{j+1}w_{k}^{\left(\beta_{1}\right)}|e_{\hat{j}-k+1}^{1}|+\sum_{k=0}^{M-j+1}w_{k}^{\left(\beta_{1}\right)}\left|e_{\hat{j}+k-1}^{1}\right|\right)+\eta_{2}\left(\sum_{k=0}^{j+1}w_{k}^{\left(\beta_{2}\right)}|e_{\hat{j}-k+1}^{1}|+\sum_{k=0}^{M-j+1}w_{k}^{\left(\beta_{2}\right)}\left|e_{\hat{j}+k-1}^{1}\right|\right)\right]\\ = & \left(1+2c_{0}^{\alpha }\left(\eta_{1}w_{1}^{\left(\beta_{1}\right)}+\eta_{2}w_{1}^{\left(\beta_{2}\right)}\right)\right)\left|e_{\hat{j}}^{1}\right|\\ & +c_{0}^{\alpha }\left[\eta_{1}\left(\sum_{k=0,k\neq 1}^{j+1}w_{k}^{\left(\beta_{1}\right)}|e_{\hat{j}}^{1}|+\sum_{k=0,k\neq 1}^{M-j+1}w_{k}^{\left(\beta_{1}\right)}\left|e_{\hat{j}}^{1}\right|\right)+\eta_{2}\left(\sum_{k=0,k\neq 1}^{j+1}w_{k}^{\left(\beta_{2}\right)}|e_{\hat{j}}^{1}|+\sum_{k=0,k\neq 1}^{M-j+1}w_{k}^{\left(\beta_{2}\right)}\left|e_{\hat{j}}^{1}\right|\right)\right]\\ \leq & \left|e_{\hat{j}}^{1}+2c_{0}^{\alpha }e_{\hat{j}}^{1}\left(\eta_{1}w_{1}^{\left(\beta_{1}\right)}+\eta_{2}w_{1}^{\left(\beta_{2}\right)}\right)\right.\\ & \left.+c_{0}^{\alpha }\left[\eta_{1}\left(\sum_{k=0,k\neq 1}^{j+1}w_{k}^{\left(\beta_{1}\right)}e_{\hat{j}-k+1}^{1}+\sum_{k=0,k\neq 1}^{M-j+1}w_{k}^{\left(\beta_{1}\right)}e_{\hat{j}+k-1}^{1}\right)+\eta_{2}\left(\sum_{k=0,k\neq 1}^{j+1}w_{k}^{\left(\beta_{2}\right)}e_{\hat{j}-k+1}^{1}+\sum_{k=0,k\neq 1}^{M-j+1}w_{k}^{\left(\beta_{2}\right)}e_{\hat{j}+k-1}^{1}\right)\right]\right|\\ = & \left|e_{\hat{j}}^{1}+c_{0}^{\alpha }\left[\eta_{1}\left(\sum_{k=0}^{j+1}w_{k}^{\left(\beta_{1}\right)}e_{\hat{j}-k+1}^{1}+\sum_{k=0}^{M-j+1}w_{k}^{\left(\beta_{1}\right)}e_{\hat{j}+k-1}^{1}\right)+\eta_{2}\left(\sum_{k=0}^{j+1}w_{k}^{\left(\beta_{2}\right)}e_{\hat{j}-k+1}^{1}+\sum_{k=0}^{M-j+1}w_{k}^{\left(\beta_{2}\right)}e_{\hat{j}+k-1}^{1}\right)\right]\right|\\ = & \tau^{\alpha }b_{0}^{\alpha }C\left(\tau^{2}+h^{2}\right)\\ \leq & \tau^{\alpha }C\left(\tau^{2}+h^{2}\right). \end{array}$$

Hence, we get

$$∥E^{1}{∥}_{\infty }\leq \tau^{\alpha }C\left(\tau^{2}+h^{2}\right).$$

Now suppose that

$$∥E^{k}∥\leq \tau^{\alpha }n^{\alpha }C\left(\tau^{2}+h^{2}\right),\;k=0,1,2,\ldots ,n.$$

Using $|v_{1}\left|-\right|v_{2}\left|\leq \right|v_{1}-v_{2}|$ and (33), it yields

$$\begin{array}{cc} & \left(1+c_{0}^{\alpha }\left[\eta_{1}\left(\sum_{k=0}^{j+1}w_{k}^{\left(\beta_{1}\right)}+\sum_{k=0}^{M-j+1}w_{k}^{\left(\beta_{1}\right)}\right)+\eta_{2}\left(\sum_{k=0}^{j+1}w_{k}^{\left(\beta_{2}\right)}+\sum_{k=0}^{M-j+1}w_{k}^{\left(\beta_{2}\right)}\right)\right]\right)\left|e_{\hat{j}}^{n+1}\right|\\ \leq & |e_{\hat{j}}^{n+1}|+c_{0}^{\alpha }\left[\eta_{1}\left(\sum_{k=0}^{j+1}w_{k}^{\left(\beta_{1}\right)}|e_{\hat{j}-k+1}^{n+1}|+\sum_{k=0}^{M-j+1}w_{k}^{\left(\beta_{1}\right)}\left|e_{\hat{j}+k-1}^{n+1}\right|\right)+\eta_{2}\left(\sum_{k=0}^{j+1}w_{k}^{\left(\beta_{2}\right)}|e_{\hat{j}-k+1}^{n+1}|+\sum_{k=0}^{M-j+1}w_{k}^{\left(\beta_{2}\right)}\left|e_{\hat{j}+k-1}^{n+1}\right|\right)\right]\\ = & \left(1+2c_{0}^{\alpha }\left(\eta_{1}w_{0}^{\left(\beta_{1}\right)}+\eta_{2}w_{1}^{\left(\beta_{2}\right)}\right)|e_{\hat{j}}^{n+1}|\right)\\ & +c_{0}^{\alpha }\left[\eta_{1}\left(\sum_{k=0,k\neq 1}^{j+1}w_{k}^{\left(\beta_{1}\right)}|e_{\hat{j}-k+1}^{n+1}|+\sum_{k=0,k\neq 1}^{M-j+1}w_{k}^{\left(\beta_{1}\right)}\left|e_{\hat{j}+k-1}^{n+1}\right|\right)+\eta_{2}\left(\sum_{k=0,k\neq 1}^{j+1}w_{k}^{\left(\beta_{2}\right)}|e_{\hat{j}-k+1}^{n+1}|+\sum_{k=0,k\neq 1}^{M-j+1}w_{k}^{\left(\beta_{2}\right)}\left|e_{\hat{j}+k-1}^{n+1}\right|\right)\right]\\ \leq & \left|e_{\hat{j}}^{n+1}+c_{0}^{\alpha }\left[\eta_{1}\left(\sum_{k=0}^{j+1}w_{k}^{\left(\beta_{1}\right)}e_{\hat{j}-k+1}^{n+1}+\sum_{k=0}^{M-j+1}w_{k}^{\left(\beta_{1}\right)}e_{\hat{j}+k-1}^{n+1}\right)+\eta_{2}\left(\sum_{k=0}^{j+1}w_{k}^{\left(\beta_{2}\right)}e_{\hat{j}-k+1}^{n+1}+\sum_{k=0}^{M-j+1}w_{k}^{\left(\beta_{2}\right)}e_{\hat{j}+k-1}^{n+1}\right)\right]\right|\\ = & |e_{\hat{j}}^{n}-\sum_{l=0}^{n-1}\left(d_{l+1}^{\alpha }-d_{l}^{\alpha }\right)\left[\eta_{1}\left(\sum_{k=0}^{j+1}w_{k}^{\left(\beta_{1}\right)}e_{\hat{j}-k+1}^{n-l}+\sum_{k=0}^{M-j+1}w_{k}^{\left(\beta_{1}\right)}e_{\hat{j}+k-1}^{n-l}\right)+\eta_{2}\left(\sum_{k=0}^{j+1}w_{k}^{\left(\beta_{2}\right)}e_{\hat{j}-k+1}^{n-l}+\sum_{k=0}^{M-j+1}w_{k}^{\left(\beta_{2}\right)}e_{\hat{j}+k-1}^{n-l}\right)\right]\\ & +\tau^{\alpha }b_{n}^{\alpha }C\left(\tau^{2}+h^{2}\right)|\\ \leq & |e_{\hat{j}}^{n}|-\sum_{l=0}^{n-1}\left(d_{l+1}^{\alpha }-d_{l}^{\alpha }\right)\left[\eta_{1}\left(\sum_{k=0}^{j+1}w_{k}^{\left(\beta_{1}\right)}|e_{\hat{j}-k+1}^{n-1}|+\sum_{k=0}^{M-j+1}w_{k}^{\left(\beta_{1}\right)}\left|e_{\hat{j}+k-1}^{n-1}\right|\right)+\eta_{2}\left(\sum_{k=0}^{j+1}w_{k}^{\left(\beta_{2}\right)}|e_{\hat{j}-k+1}^{n-1}|+\sum_{k=0}^{M-j+1}w_{k}^{\left(\beta_{2}\right)}\left|e_{\hat{j}+k-1}^{n-1}\right|\right)\right]\\ & +\tau^{\alpha }b_{n}^{\alpha }C\left(\tau^{2}+h^{2}\right)\\ = & \tau^{\alpha }n^{\alpha }C\left(\tau^{2}+h^{2}\right)-\left(d_{n}^{\alpha }-d_{0}^{\alpha }\right)\left[\eta_{1}\left(\sum_{k=0}^{j+1}w_{k}^{\left(\beta_{1}\right)}+\sum_{k=0}^{M-j+1}w_{k}^{\left(\beta_{1}\right)}\right)+\eta_{2}\left(\sum_{k=0}^{j+1}w_{k}^{\left(\beta_{2}\right)}+\sum_{k=0}^{M-j+1}w_{k}^{\left(\beta_{2}\right)}\right)\right]\tau^{\alpha }n^{\alpha }C\left(\tau^{2}+h^{2}\right)\\ & +\tau^{\alpha }b_{n}^{\alpha }C\left(\tau^{2}+h^{2}\right)\\ = & \tau^{\alpha }n^{\alpha }C\left(\tau^{2}+h^{2}\right)+\left(d_{0}^{\alpha }-d_{n}^{\alpha }\right)\left[\eta_{1}\left(\sum_{k=0}^{j+1}w_{k}^{\left(\beta_{1}\right)}+\sum_{k=0}^{M-j+1}w_{k}^{\left(\beta_{1}\right)}\right)+\eta_{2}\left(\sum_{k=0}^{j+1}w_{k}^{\left(\beta_{2}\right)}+\sum_{k=0}^{M-j+1}w_{k}^{\left(\beta_{2}\right)}\right)\right]\tau^{\alpha }n^{\alpha }C\left(\tau^{2}+h^{2}\right)\\ & +\tau^{\alpha }\left({\left(n+1\right)}^{\alpha }-n^{\alpha }\right)C\left(\tau^{2}+h^{2}\right)\\ \leq & \left(d_{0}^{\alpha }-d_{n}^{\alpha }\right)\left[\eta_{1}\left(\sum_{k=0}^{j+1}w_{k}^{\left(\beta_{1}\right)}+\sum_{k=0}^{M-j+1}w_{k}^{\left(\beta_{1}\right)}\right)+\eta_{2}\left(\sum_{k=0}^{j+1}w_{k}^{\left(\beta_{2}\right)}+\sum_{k=0}^{M-j+1}w_{k}^{\left(\beta_{2}\right)}\right)\right]\tau^{\alpha }{\left(n+1\right)}^{\alpha }C\left(\tau^{2}+h^{2}\right)\\ & +\tau^{\alpha }{\left(n+1\right)}^{\alpha }C\left(\tau^{2}+h^{2}\right)\\ = & \left(1+\left(d_{0}^{\alpha }-d_{n}^{\alpha }\right)\left[\eta_{1}\left(\sum_{k=0}^{j+1}w_{k}^{\left(\beta_{1}\right)}+\sum_{k=0}^{M-j+1}w_{k}^{\left(\beta_{1}\right)}\right)+\eta_{2}\left(\sum_{k=0}^{j+1}w_{k}^{\left(\beta_{2}\right)}+\sum_{k=0}^{M-j+1}w_{k}^{\left(\beta_{2}\right)}\right)\right]\right)\tau^{\alpha }{\left(n+1\right)}^{\alpha }C\left(\tau^{2}+h^{2}\right)\\ \leq & \left(1+\left(c_{0}^{\alpha }-d_{n}^{\alpha }\right)\left[\eta_{1}\left(\sum_{k=0}^{j+1}w_{k}^{\left(\beta_{1}\right)}+\sum_{k=0}^{M-j+1}w_{k}^{\left(\beta_{1}\right)}\right)+\eta_{2}\left(\sum_{k=0}^{j+1}w_{k}^{\left(\beta_{2}\right)}+\sum_{k=0}^{M-j+1}w_{k}^{\left(\beta_{2}\right)}\right)\right]\right)T^{\alpha }C\left(\tau^{2}+h^{2}\right). \end{array}$$

Hence

$$\begin{array}{cc} ∥E^{n+1}{∥}_{\infty }\leq & \frac{1+\left(c_{0}^{\alpha }-d_{n}^{\alpha }\right)\left[\eta_{1}\left(S^{\left(\beta_{1}\right)}+{\bar{S}}^{\left(\beta_{1}\right)}\right)+\eta_{2}\left(S^{\left(\beta_{2}\right)}+{\bar{S}}^{\left(\beta_{2}\right)}\right)\right]}{1+c_{0}^{\alpha }\left[\eta_{1}\left(S^{\left(\beta_{1}\right)}+{\bar{S}}^{\left(\beta_{1}\right)}\right)+\eta_{2}\left(S^{\left(\beta_{2}\right)}+{\bar{S}}^{\left(\beta_{2}\right)}\right)\right]}T^{\alpha }C\left(\tau^{2}+h^{2}\right)\\ \leq & T^{\alpha }C\left(\tau^{2}+h^{2}\right). \end{array}$$

Therefore, there exists a positive constant $C^{*}$ such that

$$\begin{array}{ccc} ∥E^{n+1}{∥}_{\infty } & \leq & C^{*}\left(\tau^{2}+h^{2}\right). \end{array}$$

 ☐

## 5. Numerical Experiments

In this section, some numerical experiments are given to demonstrate the effectiveness and accuracy of the proposed numerical scheme. Consider the following time-space fractional advection–diffusion equation $\left(0<\alpha <1,\;0<\beta_{1}<1,\;1<\beta_{2}\leq 2\right)$.

### Example

(35) $${}^{c}D_{t}^{\alpha }u\left(x,t\right)=\frac{\partial^{\beta_{1}}}{{\partial |x|}^{\beta_{1}}}u\left(x,t\right)+\frac{\partial^{\beta_{2}}}{{\partial |x|}^{\beta_{2}}}u\left(x,t\right)+f\left(x,t\right),\;\;\;\;\;0<x<1,0<t<1,$$

u(x,0)=0,0≤x≤1,

and the Dirichlet boundary conditions

u(0,t)=u(1,t)=0,0≤t≤1,

where

$$\begin{array}{c} f\left(x,t\right)=\left(\Gamma \left(\alpha +2\right)t+\frac{2t^{2-\alpha }}{\Gamma (3-\alpha )}\right)x^{2}{\left(1-x\right)}^{2}\\ +\frac{t+t^{2}}{\cos\left(\frac{\pi \beta_{1}}{2}\right)\Gamma \left(5-\beta_{1}\right)}\times (12\left[x^{4-\beta_{1}}+{\left(1-x\right)}^{4-\beta_{1}}\right]-6\left(4-\beta_{1}\right)\left[x^{3-\beta_{1}}+{\left(1-x\right)}^{3-\beta_{1}}\right]\\ +\left(3-\beta_{1}\right)\left(4-\beta_{1}\right)\left[x^{2-\beta_{1}}+{\left(1-x\right)}^{2-\beta_{1}}\right])\\ +\frac{t+t^{2}}{\cos\left(\frac{\pi \beta_{2}}{2}\right)\Gamma \left(5-\beta_{2}\right)}\times (12\left[x^{4-\beta_{2}}+{\left(1-x\right)}^{4-\beta_{2}}\right]-6\left(4-\beta_{2}\right)\left[x^{3-\beta_{2}}+{\left(1-x\right)}^{3-\beta_{2}}\right]\\ +\left(3-\beta_{2}\right)\left(4-\beta_{2}\right)\left[x^{2-\beta_{2}}+{\left(1-x\right)}^{2-\beta_{2}}\right]). \end{array}$$

The exact solution is $u\left(x,t\right)=\left(t+t^{2}\right)x^{2}{\left(1-x\right)}^{2}.$

**Figure 1:**
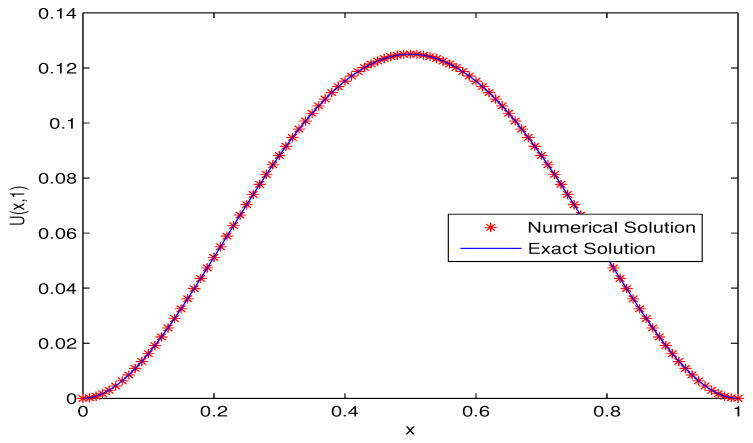
Comparison between numerical solution and exact solution for $\tau =0.01,\;h=0.01,\;\alpha =0.7,\;\beta_{1}=0.3,\;\beta_{2}=1.5,\;T=1$.

**Figure 2:**
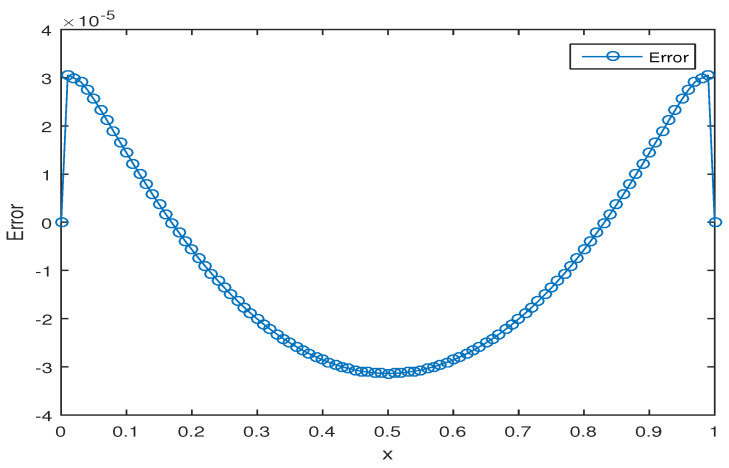
Error plot for $\tau =0.01,\;h=0.01,\;\alpha =0.7,\;\beta_{1}=0.3,\;\beta_{2}=1.5,\;T=1$.

**Figure 3:**
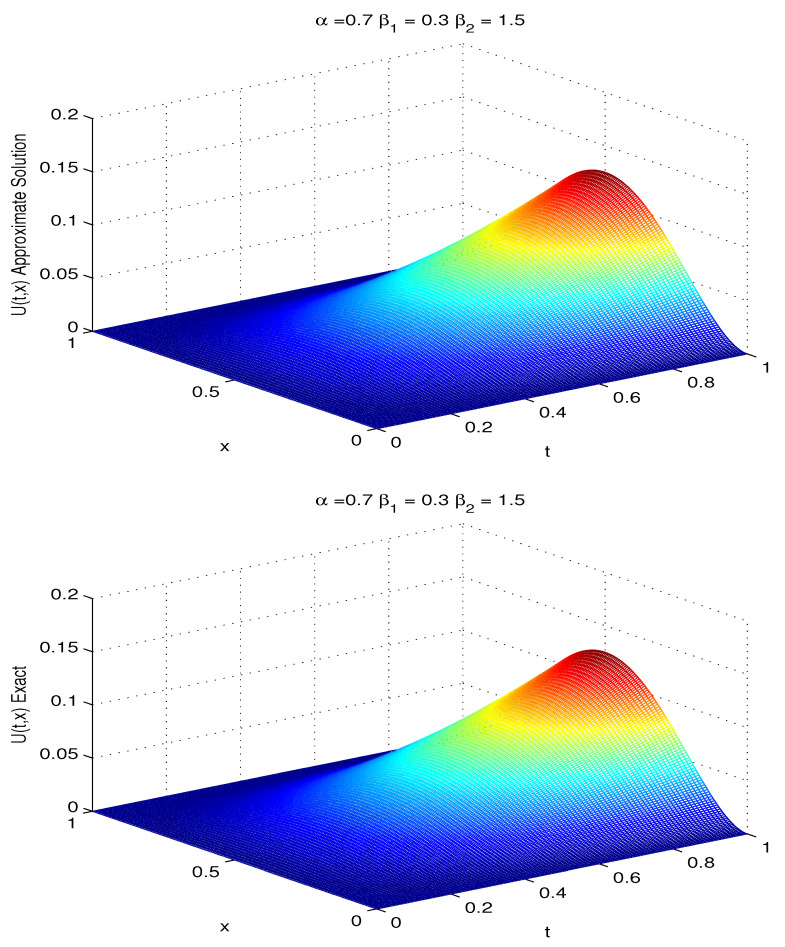
Approximate solution (**Upper**) and exact solution (**Lower**) for τ=0.01,h=0.005.

We solve this fractional advection–dispersion equation with the proposed numerical scheme with $K_{\beta_{1}}=K_{\beta_{2}}=1,\;T=1,\;L=1$. The comparison of the numerical solution with the exact solution for h=τ=0.01, $\alpha =0.7,\;\beta_{1}=0.3,\;\beta_{2}=1.5$ at T=1 is given in Figure 1. To compare the numerical and the exact solutions, error plot is given in Figure 2. For fixed $\alpha =0.7,\;\beta_{1}=0.3,\;\beta_{2}=1.5$ the exact solution and the approximate solution with temporal and spatial steps τ=0.01,h=0.005 are shown in Figure 3.

The spatial errors and convergence orders of the proposed scheme for solving (35) are shown in Table 1 and Table 2 for different values of $\beta_{1}$ and $\beta_{2}$, respectively. Fixing $\tau =0.01,\;\alpha =0.9,\;\beta_{2}=1.6$. The $L_{2}$–norm is used to compute the error of the numerical solution at the last time step by

$$E\left(\tau ,h\right)=\sqrt{h\sum_{j=1}^{M-1}{\left|u\left(x_{j},t_{N}\right)-u_{j}^{N}\right|}^{2}}.$$

Next, we fix the spatial step size h=0.01 and vary the time step. Table 3 presents the errors and convergence order for various values of α at time T=1. The numerical convergence order in the spatial and temporal direction is $O(\tau^{2}+h^{2})$, as in Theorem 2.

## 6. Conclusions

In this article, we have proposed a finite difference method for solving a class of time-space fractional advection–dispersion equation. We combined the trapezoidal formula, which is well known for the numerical integration of Riemann–Liouville integral, with the Grünwald–Letnikov discretization of the Riesz fractional derivative in space to obtain a numerical scheme. We proved that our proposed scheme is stable and convergent with the accuracy of $O(\tau^{2}+h^{2})$ under the sufficient conditions $2^{\alpha +1}\leq 3$ and $\frac{\sqrt{17}-1}{2}\leq \beta_{2}\leq 2.$ However, our numerical experiments given in Table 2 and Table 3 depict that when $2^{\alpha +1}>3$ and $\beta_{2}<\frac{\sqrt{17}-1}{2}$ the presented numerical method is still stable and convergent for various temporal and spatial time steps. Finally, some numerical experiments for the fractional finite difference method are given that agree very well with our theoretical results.

## Figures and Tables

**Table 1:** The errors and convergence order when τ=0.01, $\alpha =0.9,\;\beta_{2}=1.6,\;T=1$.

$\beta_{1}=0.1$			$\beta_{1}=0.3$		$\beta_{1}=0.5$		$\beta_{1}=0.7$	
h	E(τ,h)	**order**	E(τ,h)	**order**	E(τ,h)	**order**	E(τ,h)	**order**
1/10	2.3743 $\times \;10^{-3}$	-	2.4068 $\times \;10^{-3}$	-	2.4513 $\times \;10^{-3}$	-	2.5127 $\times \;10^{-3}$	-
1/20	5.7815 $\times \;10^{-4}$	2.0380	5.8594 $\times \;10^{-4}$	2.0382	5.9700 $\times \;10^{-4}$	2.0377	6.1222 $\times \;10^{-4}$	2.0371
1/40	1.4044 $\times \;10^{-4}$	2.0414	1.4235 $\times \;10^{-4}$	2.0413	1.4512 $\times \;10^{-4}$	2.0404	1.4893 $\times \;10^{-4}$	2.0393
1/80	3.3971 $\times \;10^{-5}$	2.0475	3.4438 $\times \;10^{-5}$	2.0473	3.5130 $\times \;10^{-5}$	2.0464	3.6083 $\times \;10^{-5}$	2.0452
1/160	7.9725 $\times \;10^{-6}$	2.0912	8.0845 $\times \;10^{-6}$	2.0907	8.2539 $\times \;10^{-6}$	2.0895	8.4892 $\times \;10^{-6}$	2.0876

**Table 2:** The errors and convergence order when τ=0.01, $\alpha =0.7,\;\beta_{1}=0.3,\;T=1$.

$\beta_{2}=1.4$			$\beta_{2}=1.6$		$\beta_{2}=1.8$		$\beta_{2}=2$	
h	E(τ,h)	**order**	E(τ,h)	order	E(τ,h)	**order**	E(τ,h)	**order**
1/10	2.1509 $\times \;10^{-3}$	-	2.4462 $\times \;10^{-3}$	-	2.7484 $\times \;10^{-3}$	-	2.9426 $\times \;10^{-3}$	-
1/20	5.4062 $\times \;10^{-4}$	1.9923	5.9481 $\times \;10^{-4}$	2.0400	6.6725 $\times \;10^{-4}$	2.0422	7.3624 $\times \;10^{-4}$	1.9988
1/40	1.3605 $\times \;10^{-4}$	1.9904	1.4434 $\times \;10^{-4}$	2.0429	1.6095 $\times \;10^{-4}$	2.0516	1.8368 $\times \;10^{-4}$	2.0029
1/80	3.4175 $\times \;10^{-5}$	1.9931	3.4894 $\times \;10^{-5}$	2.0484	3.8429 $\times \;10^{-5}$	2.0663	4.5457 $\times \;10^{-5}$	2.0146
1/160	8.3926 $\times \;10^{-6}$	2.0257	8.1970 $\times \;10^{-6}$	2.0898	8.8282 $\times \;10^{-6}$	2.1220	1.0893 $\times \;10^{-5}$	2.0609

**Table 3:** The errors and convergence order when h=0.001, $\beta_{1}=0.5,\;\beta_{2}=1.4,\;T=1$.

α=0.2			α=0.4		α=0.6		α=0.8	
t	E(τ,h)	**order**	E(τ,h)	**order**	E(τ,h)	**order**	E(τ,h)	**order**
1/5	1.4377 $\times \;10^{-4}$	-	2.0772 $\times \;10^{-4}$	-	2.3616 $\times \;10^{-4}$	-	2.5162 $\times \;10^{-5}$	-
1/10	3.8544 $\times \;10^{-5}$	1.8992	5.3402 $\times \;10^{-5}$	1.9596	5.9403 $\times \;10^{-5}$	1.9911	6.2750 $\times \;10^{-5}$	2.0035
1/20	1.0120 $\times \;10^{-5}$	1.9293	1.3551 $\times \;10^{-5}$	1.9784	1.4831 $\times \;10^{-5}$	2.0018	1.5587 $\times \;10^{-5}$	2.0092
1/40	2.5497 $\times \;10^{-6}$	1.9888	3.3379 $\times \;10^{-6}$	2.0214	3.6155 $\times \;10^{-6}$	2.0363	3.7925 $\times \;10^{-6}$	2.0391
1/80	5.7769 $\times \;10^{-7}$	2.1419	7.5305 $\times \;10^{-7}$	2.1481	8.1450 $\times \;10^{-7}$	2.1502	8.5804 $\times \;10^{-7}$	2.1440
